# The accumulation of modular serine protease mediated by a novel circRNA sponging miRNA increases *Aedes aegypti* immunity to fungus

**DOI:** 10.1186/s12915-024-01811-6

**Published:** 2024-01-17

**Authors:** Tengfei Lu, Yannan Ji, Mengmeng Chang, Xiaoming Zhang, Yanhong Wang, Zhen Zou

**Affiliations:** 1 State Key Laboratory of Integrated Management of Pest Insects and Rodents, Institute of Zoology, Chinese Academy of Sciences, Beijing, 100101 China; 2 CAS Center for Excellence in Biotic Interactions, University of Chinese Academy of Sciences, Beijing, 100049 China

**Keywords:** miRNA, ModSP, Antifungal immunity, *Beauveria bassiana*, *Aedes aegypti*

## Abstract

**Background:**

Mosquitoes transmit many infectious diseases that affect human health. The fungus *Beauveria bassiana* is a biological pesticide that is pathogenic to mosquitoes but harmless to the environment.

**Results:**

We found a microRNA (miRNA) that can modulate the antifungal immunity of *Aedes aegypti* by inhibiting its cognate serine protease. Fungal infection can induce the expression of modular serine protease (ModSP), and ModSP knockdown mosquitoes were more sensitive to *B. bassiana* infection. The novel miRNA-novel-53 is linked to antifungal immune response and was greatly diminished in infected mosquitoes*.* The miRNA-novel-53 could bind to the coding sequences of *ModSP* and impede its expression. Double fluorescence in situ hybridization (FISH) showed that this inhibition occurred in the cytoplasm. The amount of miRNA-novel-53 increased after miRNA agomir injection. This resulted in a significant decrease in ModSP transcript and a significant increase in mortality after fungal infection. An opposite effect was produced after antagomir injection. The miRNA-novel-53 was also knocked out using CRISPR-Cas9, which increased mosquito resistance to the fungus *B. bassiana*. Moreover, mosquito novel-circ-930 can affect *ModSP* mRNA by interacting with miRNA-novel-53 during transfection with siRNA or overexpression plasmid.

**Conclusions:**

Novel-circ-930 affects the expression level of ModSP by a novel-circ-930/miRNA-novel-53/*ModSP* mechanism to modulate antifungal immunity, revealing new information on innate immunity in insects.

**Supplementary Information:**

The online version contains supplementary material available at 10.1186/s12915-024-01811-6.

## Highlights

• *ModSP* in *Aedes aegypti* is upregulated upon *Beauveria bassiana* infection.

• *ModSP* positively regulates mosquito immune responses.

• *ModSP* is a target of miRNA-novel-53.

• miRNA-novel-53 level is downregulated after fungal infection.

• miRNA-novel-53 negatively regulates mosquito antifungal immunity.

• novel-circ-930 acts as a miRNA-novel-53 sponge.

**Supplementary Information:**

The online version contains supplementary material available at 10.1186/s12915-024-01811-6.

## Background

Human health can be threatened by mosquito-transmitted diseases [1–3]. *Aedes aegypti* is the main mosquito vector of arboviruses such as dengue, yellow fever, Zika, and chikungunya viruses. Control of vector mosquitoes is essential for the control of vector-borne diseases. Excessive use of chemical insecticides has led to resistant mosquito populations and environmental pollution. Entomopathogenic fungi, such as *Beauveria bassiana*, *Cordyceps militaris*, and *Metarhizium anisopliae*, are environmentally safe biological control agents that are usually harmless to nontarget species [4–6].

Serine protease (SP) has important functions in many biological processes, including insect innate immunity. SPs usually exist in the form of inactive zymogens. The downstream proteases in the cascade are not activated until the upstream ones are activated. *Drosophila* modular serine protease (ModSP), *Tenebrio molitor* modular serine protease (MSP), and *Manduca sexta* hemolymph proteinase 14 (HP14) are activated after the specific recognition by pathogen-associated molecular patterns (PAMPs). They act as the initiating protease in their respective immune pathways, especially prophenoloxidase (PPO) activation and the production of AMPs via the Toll signaling pathway [7, 8]. *Drosophila* ModSP can be activated by the gram-negative bacteria-binding protein 3 (GNBP3) and peptidoglycan recognition protein (PGRP-SA) recognition complex [9]. ModSP is recruited by pattern recognition receptors (PRRs) to increase its local concentration and achieve a level of autoactivation [9]. Similarly, *M. sexta* proHP14 can be activated in response to fungal infection by the recognition of β-1,3-glucan recognition protein 1 (βGRP1) [10] or βGRP2 [11]. The interaction of the LDLa_2-5_ region of proHP14 and microbial binding protein (MBP) C-terminal domain and the binding of DAP-PG by PGRP1 and MBP N-terminal domain have been found to be necessary for proHP14 autoactivation by DAP-type peptidoglycan. These interactions lead to autoactivation by concentrating proHP14 proenzyme on the surface of invading microorganisms [12].

MicroRNAs (miRNAs), usually 19–24 nt in length, are single-stranded noncoding RNA encoded by endogenous genes. miRNAs are common in eukaryotes and regulate target genes at both transcriptional and posttranscriptional levels [13, 14]. miRNAs are involved with almost all biological processes, including development, metabolism, cell differentiation, reproduction, and immunity [15–19]. The earliest discovered miRNA was found in *Caenorhabditis elegans* in the 1990s [20]. This miRNA, named lin-4, regulates lin-14 and affects development [20]. The first insect miRNA was recognized in *Drosophila melanogaster* in 2001 [21]. In recent years, miRNAs have been identified and characterized in many insects using high-throughput small RNA sequencing technologies.

miRNAs are indispensable in the posttranscriptional regulation involved in insect immunity [22–24]. The expression levels of antimicrobial peptides (AMPs) increased in miR-8 knockout *D. melanogaster* under normal physiological conditions. An abnormal level of AMPs in miR-8-deficient *D. melanogaster* was found after the introduction of miR-8. This indicated that *Drosophila* miR-8 is important in balancing immunity [25]. *Drosophila* let-7 was also found to inhibit the translation of AMPs [26]. *Drosophila* miR-317 can directly bind the 3′ UTR of *Dif-Rc*, lowering the amount of AMP *Drs* and suppressing the Toll pathway. The miRNA-317 overexpressing flies had high mortality after *Micrococcus luteus* infection, but survival was better in miR-317-deficient *D. melanogaster* than that in the control group. It was postulated that miR-317 is important for *Drosophila* survival and innate immunity [27]. Changes in the abundance of host miRNA following infection suggest that miRNAs might directly target the virus genes. *Bombyx mori* miR-8 inhibits several *Bombyx mori* nucleopolyhedrovirus (BmNPV) genes, and blockage of the miRNA produced a significant increase in viral titer in the fat bodies of infected larvae [28]. Additionally, an insect defense strategy, utilizing miRNA to silence pathogen-related genes, was discovered. Host let-7 and miR-100 were transported into *B. bassiana* with the specific function of inhibiting the virulence-associated factors, *sec2p* and *C6TF*, conferring resistance to infection [29].

Circular RNAs (circRNAs) are a distinct class of endogenous single-stranded noncoding RNA generated by back-splicing process without a cap structure at the 5′ end and a polyadenylated tail at the 3′ end. They were considered as splicing errors without potential function in earlier research [30, 31]. However, it was found that circRNAs are conserved throughout evolutionary processes, and they have spatiotemporal expression specificity, indicating that circRNAs may have regulatory functions [32–34]. High-throughput RNA sequencing was used to study the differentially expressed RNAs in the fat body of *B. mori* infected with BmNPV. The circRNA/miRNA/mRNA regulatory network was constructed, indicating that silkworm circRNA can act as molecular sponges of miRNAs to participate in the immune response against BmNPV [35]. *Cdr1as* can bind miR-7, facilitating a specific miR-7-AGO2 interaction, and the activity of miR-7 was inhibited in diseases including diabetes and colorectal cancer [32, 36].

We found that *Ae. aegypti* ModSP significantly increased after infection with *B. bassiana*. Mosquitoes became more susceptible to the entomopathogenic fungus after knocking down *ModSP*. Additionally, a new miRNA, miRNA-novel-53, was found to inhibit *ModSP* by directly binding to its coding sequence (CDS) region in the cytoplasm. This increased mortality during *B. bassiana* infection. CRISPR-Cas9 knockout of miRNA-novel-53 increased mosquito resistance to *B. bassiana*. Mosquito novel-circ-930 can regulate *ModSP* mRNA by interacting with miRNA-novel-53 according to cell transfection with siRNA or overexpression vector. These findings show that miRNA-novel-53 and *ModSP* cooperate to modulate antifungal immunity and provide insights on the regulatory mechanisms of innate immunity in insects.

## Results

### ModSP responses to fungal infection in *Ae. aegypti*

**Fig. 1:**
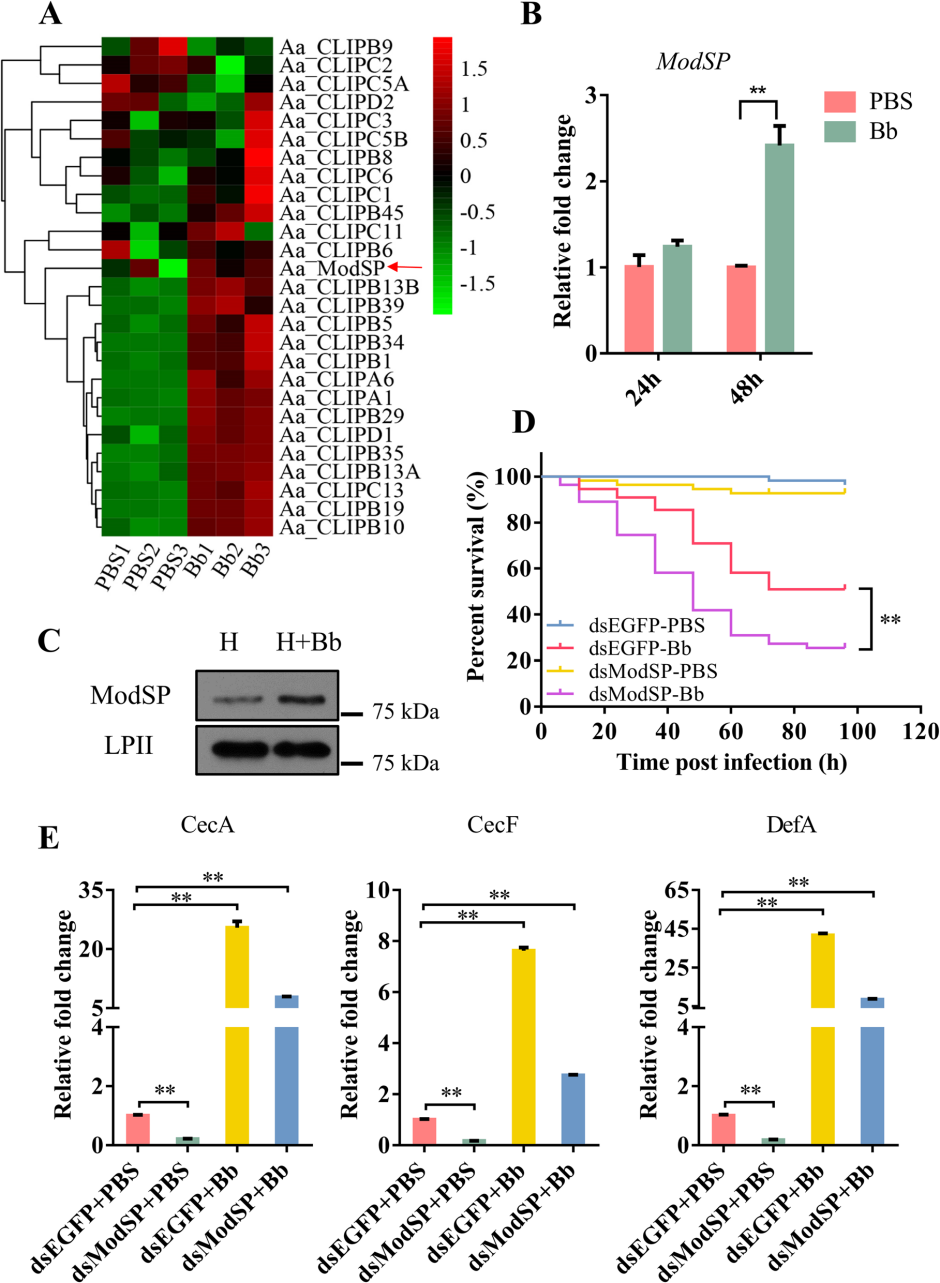
ModSP responses to fungal infection in *Aedes aegypti*. **A** Hierarchical clustering analysis of different expressive genes of serine proteases in mosquitoes infected with *Beauveria bassiana* for 48 h. PBS, mosquitoes treated with PBS; Bb, mosquitoes infected with *B. bassiana*. **B** qRT-PCR examination of transcript levels of *ModSP* after *B. bassiana* treatment. The qRT-PCR results were normalized to the internal control rps7. The results were given in triplicate and presented as mean ± SEM. The Student’s *t*-test was used to determine the statistical significance of treatment means (GraphPad Prism 6). **P < 0.01. PBS, mosquitoes treated with PBS; Bb, mosquitoes infected with *B. bassiana*. **C** Expression levels of ModSP protein were evaluated by immunoblotting of *Ae. aegypti* hemolymph using the ModSP polyclonal antibody. Lipophorin II (LPII) was used as the loading control. H, hemolymph of mosquitoes treated with PBS for 48 h; H + Bb, hemolymph of mosquitoes infected with *B. bassiana* for 48 h. **D** The survival status of mosquitoes infected with *B. bassiana* was additionally aggravated by *ModSP* RNAi treatment (dsModSP + Bb). Three repetitions of each experiment were carried out. The Kaplan-Meier approach was used to draw the survival curves, and the *P*-value was defined by the log-rank (Mantel-Cox) test. **P < 0.01. dsEGFP + PBS, dsEGFP + Bb, dsEGFP-injected mosquitoes treated with PBS or *B. bassiana* conidia; dsModSP + PBS, dsModSP + Bb, dsModSP-injected mosquitoes treated with PBS or *B. bassiana* conidia. **E** Expression levels of *AMPs* in mosquitoes treated with PBS or *B. bassiana* conidia for 48 h. The qRT-PCR results were normalized to the internal control rps7. The results were fulfilled in triplicate and are presented as mean ± SEM. The Student’s *t*-test was used to compare the statistical significance of treatment means (GraphPad Prism 6). **P < 0.01

We previously demonstrated that *B. bassiana* infection can significantly decrease mosquito survival rate [37, 38]. To investigate the function of SPs in antifungal immunity, RNA-Seq was employed to determine the expression patterns of SPs after fungal infection (Additional file 1: Table S1). Phylogenetic analysis was performed using key SPs/SPHs from the sequencing data based on serine protease cascades in multiple species (Additional file 2: Figure S1A, Additional file 3: Table S2). This analysis demonstrated that the SPs/SPHs belong to different clusters. *Aedes aegypti* ModSP belongs to cluster V and is homologous to *M. sexta* HP14 [39]. To view the expression patterns of these immunity-related SP genes, we generated a heat map based on their level of gene expression. The genes associated with the melanization cascades were mostly upregulated 48 h after fungal infection, with *ModSP* moderately upregulated (Fig. 1A). Quantitative real-time PCR (qRT-PCR) analysis indicated a significant increase in the expression levels of *CLIPC1*, *CLIPB8*, and *CLIPB5* in fungi-infected mosquitoes (Additional file 2: Figure S1B). These are important enzymes in the predicted SP cascade in *Ae. aegypti*. The expressions of *CLIPB5*, *CLIPB10*, and *CLIPB29* markedly increased, which was consistent with the sequencing analysis and results from a previous study [40]. Also, the expression level of *Ae. aegypti ModSP* mRNA was detected at 6, 12, 24, and 48 h after infection, and it increased to more than 2.2-fold at 48 h (Fig. 1B, Additional file 2: Figure S1C). An immunoblot showed that the production of ModSP was elevated in the hemolymph of infected mosquitoes at 48 h (Fig. 1C).

To evaluate the role of ModSP in immune responses, we studied the vulnerability of interfered *ModSP* mosquitoes to *B. bassiana* infection. The efficiency of knockdown of *ModSP* was inspected at the mRNA level by qRT-PCR. The level was significantly downregulated in adults after 3 days, compared to the control EGFP RNAi treatment (Additional file 2: Figure S1D). Mosquitoes were infected by *B. bassiana* 3 days after receiving ModSP dsRNA injection, and their mortality was scored. The mortality of infected mosquitoes was also reduced by ModSP RNAi depletion (dsModSP + Bb) compared to EGFP RNAi depletion (dsEGFP + Bb) (Fig. 1D). In addition, the mRNA levels of several *AMPs*, such as *cecropin A* (*CecA*), *cecropin F* (*CecF*), and *defensin A* (*DefA*) significantly increased after *B. bassiana* infection for 48 h (Fig. 1E). However, the expression levels of these *AMPs* decreased after injection with *ModSP* dsRNA (Fig. 1E). Thus, knockdown of ModSP in *Ae. aegypti* resulted in lower resistance to *B. bassiana* infection and indicated that ModSP is important in the antifungal response.

### Analysis of miRNA response to *B. bassiana* infection in *Ae. aegypti*

**Fig. 2:**
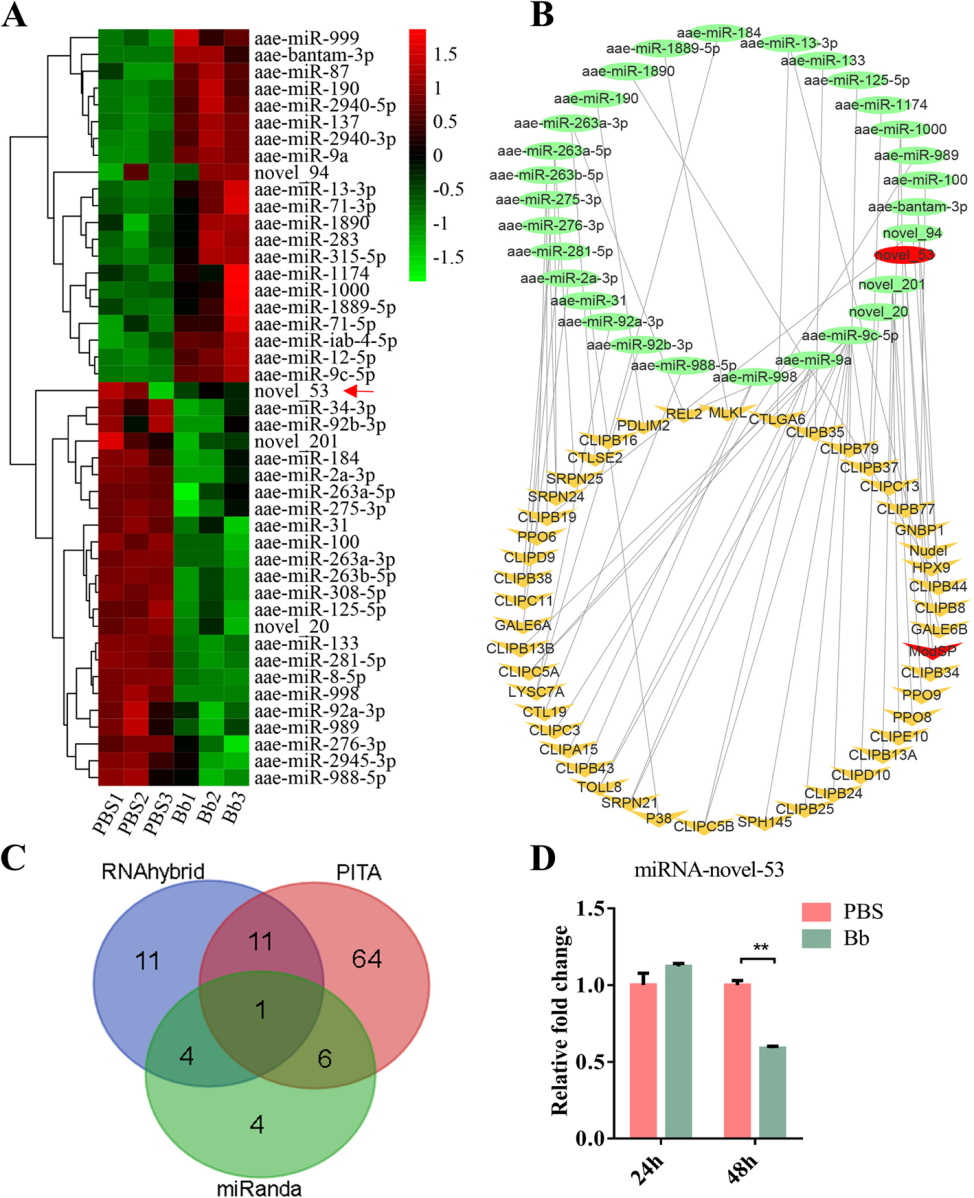
Analysis of miRNA response to *B. bassiana* infection in *Ae. Aegypti*. **A** Hierarchical clustering analysis of miRNAs with different expression levels in mosquitoes infected with *B. bassiana*. PBS, mosquitoes treated with PBS; Bb, mosquitoes infected with *B. bassiana*. **B** miRNA/mRNA regulatory networks according to an intersection of the three software programs in *Ae. aegypti*. Ellipses indicate miRNAs, and V shapes indicate target mRNAs. **C** Venn diagram depicting the overlap of a total of 101 miRNAs targeting *ModSP* predicted by three pieces of software tools: PITA, miRanda, and RNAhybrid. **D** qRT-PCR analysis of transcript levels of miRNA-novel-53 after *B. bassiana* infection. The qRT-PCR results were normalized to the internal control U6. The results were obtained in triplicate and are presented as mean ± SEM. The Student’s *t*-test was utilized to calculate the significance of treatment mean differences. *P < 0.05; **P < 0.01. PBS, mosquitoes treated with PBS; Bb, mosquitoes infected with *B. bassiana*

miRNAs are involved in almost all biological processes of insects, including innate immunity, through posttranscriptional mechanisms. To investigate whether miRNAs participate in regulating the differential expression of *ModSP*, we used high-throughput small RNA sequencing to study the expression profiles of miRNAs after fungal infection. We used the miREvo and the miRDeep2 algorithm packages to find 122 identified miRNAs and 68 new miRNAs in the clean data obtained by small RNA (sRNA) sequencing based on the trait of the miRNA precursor (Additional file 4: Table S3). Transcripts per million (TPM) was calculated to normalize the miRNA abundance. All miRNAs used for expression level analysis had a TPM greater than 50 in any group. Differentially expressed miRNAs (DEMs) between two groups were filtered by fold change and adjusted *P*-value (padj < 0.05). In mosquitoes infected with *B. bassiana*, 21 DEMs were upregulated, whereas 24 DEMs were substantially downregulated, compared to control groups (Fig. 2A, Additional file 5: Table S4). Among these DEMs, one newly discovered miRNA was upregulated, and two newly discovered miRNAs were downregulated.

We conducted target mRNA predictions on all the discovered and novel miRNAs to search for significant miRNAs and their related functional genes with important roles in *Ae. aegypti* immunity. Some DEMs and corresponding target genes were also analyzed. miRNA/mRNA regulatory networks were built using a combination of three pieces of software tools: PITA, miRanda, and RNAhybrid (Fig. 2B)*.* The genes with immunological function carry potential target sites of at least one miRNA, which suggests that the miRNAs perform an important function in immunomodulation. A total of 101 miRNAs targeting *ModSP* were found using the three pieces of software tools mentioned above. Only one miRNA, miRNA-novel-53, was detected by all the three tools as shown in the Venn diagram (Fig. 2C). These results suggest that multiple miRNAs can respond to fungal infection in *Ae. aegypti*. The existence of the new miRNA-novel-53 precursor was verified by sequencing (Additional file 2: Figure S2A). Hairpin structure prediction of the miRNA-novel-53 precursor was completed by using RNAstructure 6.3 software (Additional file 2: Figure S2B). The length of mature miRNA-novel-53 was found by Northern blot, which characterized the authenticity of the novel miRNA in different samples (Additional file 2: Figure S2C). The qRT-PCR results showed that miRNA-novel-53 was reduced to about 60% 2 days after infection with *B. bassiana* (Fig. 2D). Compared with the quantitative analysis of *ModSP*, the expression level change was in the opposite direction.

### Validation of potential targeting relationship between miRNA-novel-53 and *ModSP*

**Fig. 3:**
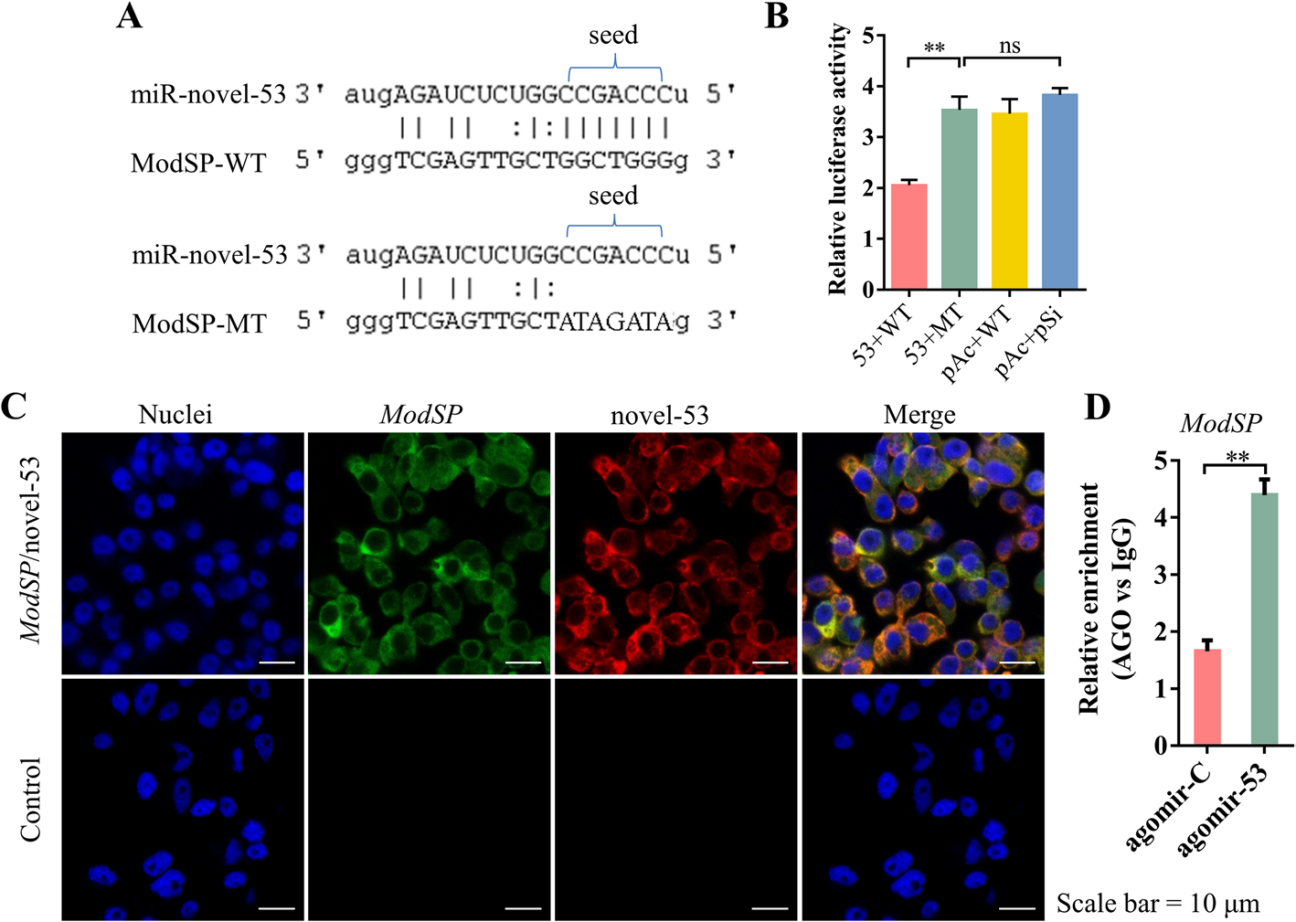
Validation of potential targeting relationship between miRNA-novel-53 and *ModSP*. **A** Sequence alignment of miRNA-novel-53 and the predicted binding site in *ModSP*. ModSP-WT, predicted target sites in *ModSP*; ModSP-MT, the binding sites of *ModSP* were mutated into noncomplementary bases with the miRNA-novel-53 seed sequence. **B** Relative luminescence activity after transfection of different plasmids in the S2 cell line. 53 + WT, cells transfected with pAc5.1b-miRNA-novel-53 and psiCHECK2-ModSP-WT plasmids; 53 + MT, cells transfected with pAc5.1b-miRNA-novel-53 and psiCHECK2-ModSP-MT plasmids; pAc + WT, cells transfected with pAc5.1b empty vector and psiCHECK2-ModSP-WT plasmid; pAc + pSi, cells transfected with pAc5.1b and psiCHECK2 plasmids. Each sample had in four replicates. Statistical differences between sample means were determined using the Student’s *t*-test. ns, *P* > 0.05; **P < 0.01. **C** Localization of miRNA-novel-53 and *ModSP* was detected with FISH in Aag2 cells. Green, *ModSP*; red, miRNA-novel-53; blue, Hoechst 33342. Scale bar, 10 μm. **D** Relative abundance of precipitated *ModSP* mRNA by *Ae. aegypti* AGO1 RIP in the different samples. **P < 0.01

The targeting relationship between miRNAs and mRNAs predicted by multiple algorithms suggested that miRNA-novel-53 targets and regulates the expression of *ModSP*. We found that the potential target site of miRNA-novel-53 is located in the CDS region of *ModSP*. A modified *ModSP*-binding sequence, which was mutated into noncomplementary bases with the miRNA-novel-53 seed region, acted as a negative control. To confirm this targeting relationship, we constructed the expression vector of miRNA-novel-53 and the double luciferase reporter gene detection vector (Fig. 3A). The luciferase activity in S2 cells in the presence of miRNA-novel-53 and *ModSP* CDS sequence decreased to nearly 55%, but the activity did not change when the *ModSP* seed region was mutated (Fig. 3B). These findings showed that miRNA-novel-53 can inhibit *ModSP* expression by binding to its CDS region.

**Fig. 4:**
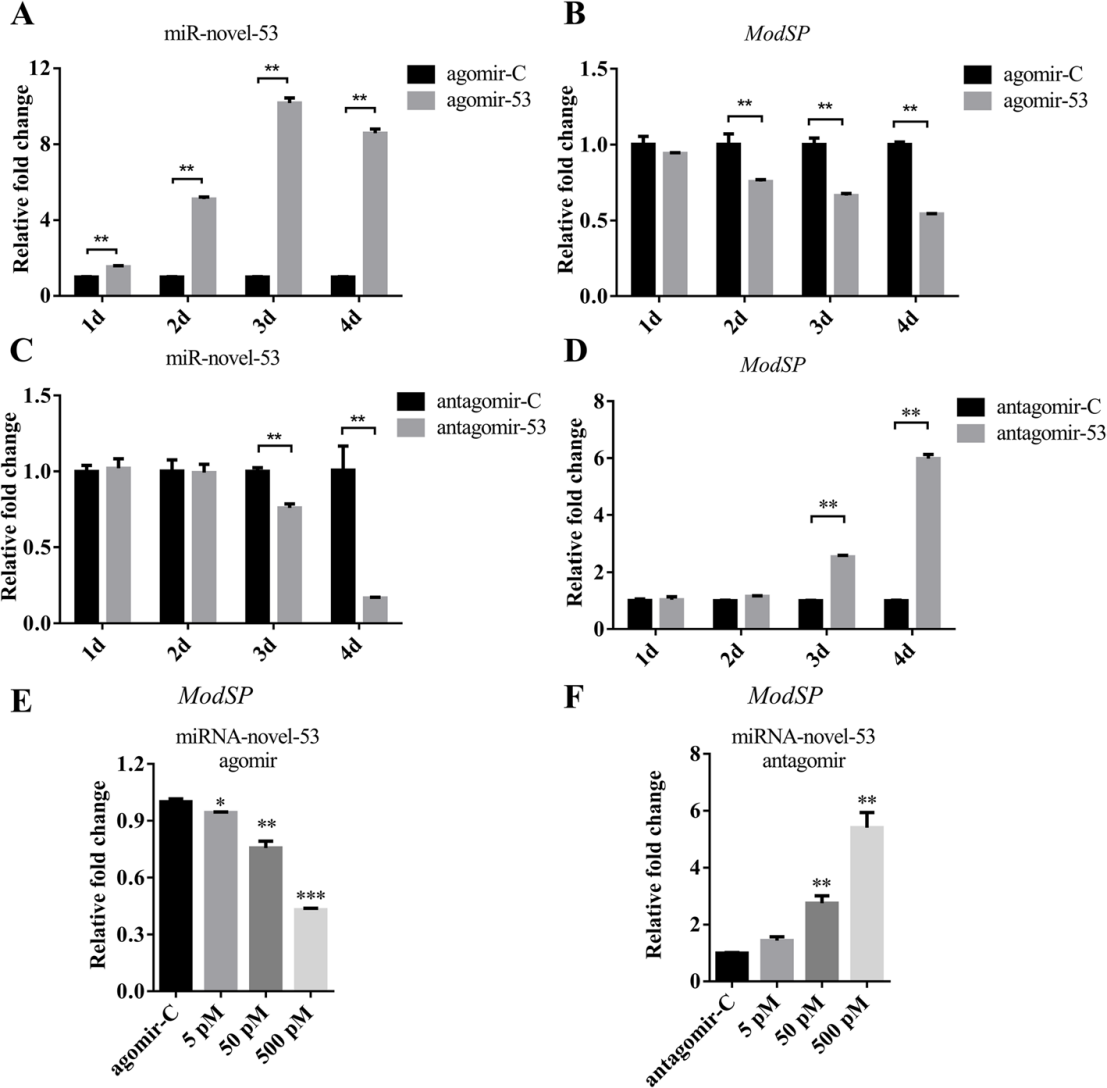
Effect of miRNA-novel-53 administration on mRNA expression of *ModSP* in* Ae. Aegypti*. The relative miRNA abundance of miR-novel-53 in *Ae. aegypti* after injection with agomir-53 (**A**) or antagomir-53 (**C**). The relative mRNA abundance of *ModSP* in *Ae. aegypti* after injection with agomir-53 (**B**) or antagomir-53 (**D**). Intrathoracic inoculation of a serial concentrations of either miRNA-novel-53 agomir (**E**) or antagomir (**F**) changed *ModSP* expression in *Ae. aegypti*. Mosquitoes were injected with 5-pM, 50-pM, and 500-pM agomir or antagomir. The results were measured in triplicate and are shown as mean ± SEM. The significance of treatment differences was determined with the Student’s *t*-test. *P < 0.05, **P < 0.01

To determine the cell location where miRNA-novel-53 inhibits the expression of *ModSP*, double FISH was performed in Aag2 cells. The images showed that both miRNA-novel-53 and *ModSP* mRNA were both mainly detected in the cytoplasm (Fig. 3C), and it suggests that regulation likely occurs in the cytoplasm. To study the interaction between miR-novel-53 and *ModSP *in vivo, an RNA immunoprecipitation (RIP) experiment was conducted using the antibody against mosquito AGO1. Injection of agomir-53 significantly increased the abundance of miR-novel-53 in mosquitoes (Fig. 4A). This led to a notable enrichment of *ModSP* mRNA in the AGO1-immunoprecipitated RNAs (Fig. 3D). Therefore, miR-novel-53 can target and interact with *ModSP *in vitro and in vivo.

### miR-novel-53 regulated the expression of *ModSP* and *AMP* in vivo

The relative expression levels of *ModSP* and miRNA-novel-53 at different developmental stages of larvae were determined. The expression level of *ModSP* increased with larval growth (Additional file 2: Figure S3A), while the expression level of the miRNA significantly decreased (Additional file 2: Figure S3B). The relative expression levels of *AMPs* were also investigated. The expression level of *CecA*, *DefA*, and *CecF* increased with the developmental stage (Additional file 2: Figure S3C–E). The relative abundance of *ModSP* and the miRNA in different tissues of adult *Ae. aegypti* 48 h after infection was also measured. The transcription level of *ModSP* significantly increased in the head, fat body, and midgut of adult *Ae. aegypti* (Additional file 2: Figure S3F). The relative expression of miRNA-novel-53 significantly decreased in the three adult tissues (Additional file 2: Figure S3G). There was no apparently change in the relative expression level of *ModSP* and miRNA-novel-53 in the Malpighian tubules and ovary.

To study the effect of miRNA-novel-53 administration on the expression level of *ModSP*, we inhibited the miRNA by injecting antagomir-53 and overexpressed it by injecting agomir-53 into the thorax of female mosquitoes within 1-d post-eclosion. The relative expression level of miRNA-novel-53 significantly increased after the agomir-53 treatment. After 3 days, the level was about 10 times higher than the level in the control group (Fig. 4A). The expression level of *ModSP* decreased significantly 2 days after agomir-53 injection, and its relative expression further decreased with time (Fig. 4B). However, antagomir-53 injection lowered the expression level of miRNA-novel-53. The expression level significantly decreased 3 days after antagomir treatment, and it decreased to less than 20% after 4 days (Fig. 4C). Corresponding to the above result, the transcription level of *ModSP* significantly increased to around eight times the initial level (Fig. 4D). Intrathoracic inoculation of a serial concentration of either miRNA-novel-53 agomir (Fig. 4E) or antagomir (Fig. 4F) led to a decrease (agomir) or an increase (antagomir) of *ModSP* in a dose-dependent manner. These results showed that the changes in miRNA-novel-53 and *ModSP* expression levels occurred in opposite directions. Therefore, miRNA-novel-53 may be involved in regulating the expression of *ModSP*.

### miRNA-novel-53 modulates the expression of ModSP which participates in antifungal immunity

**Fig. 5:**
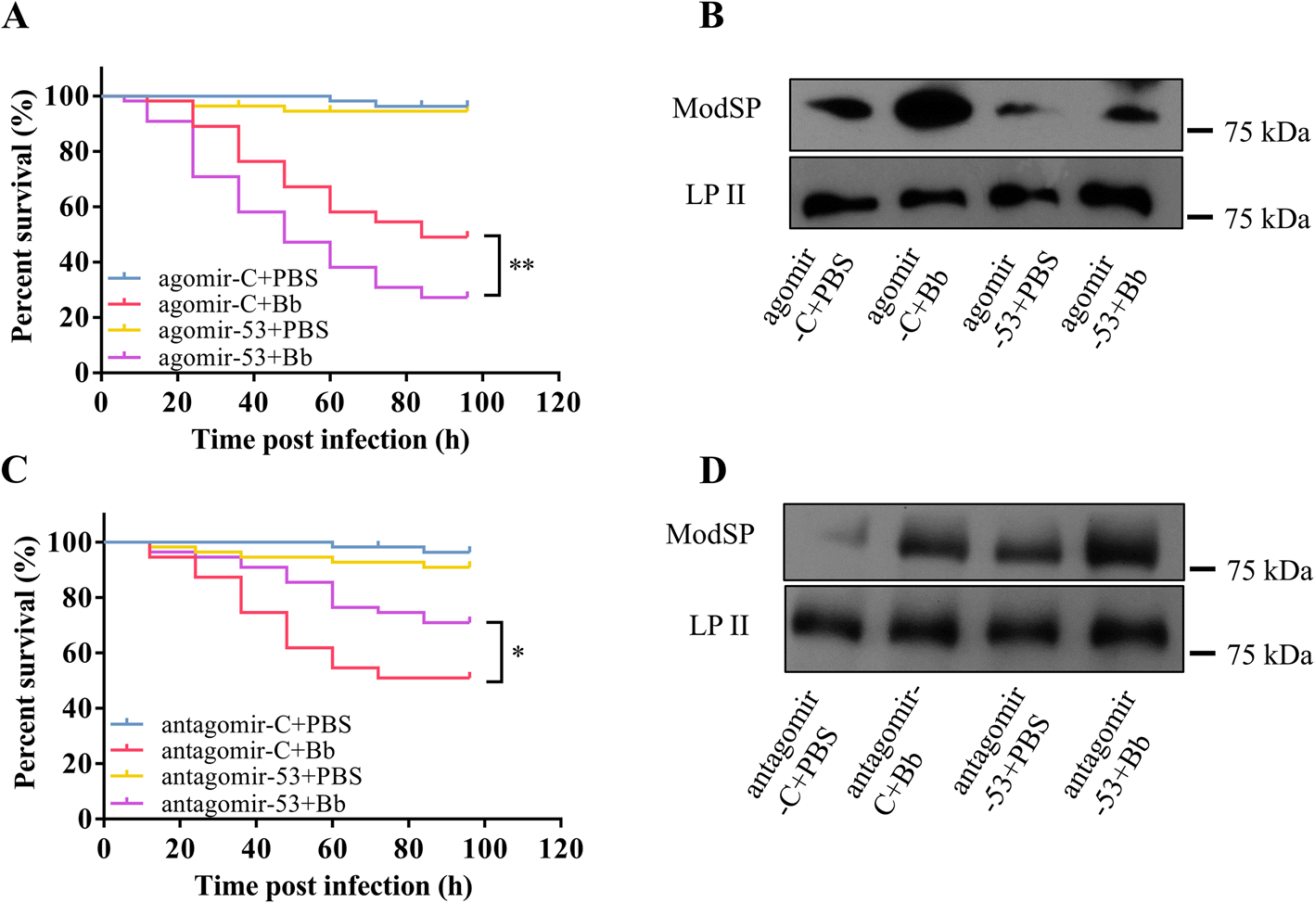
miRNA-novel-53 modulates the expression of *ModSP* that participates in antifungal immunity of *Ae. Aegypti*. The survival rate of *Ae. aegypti* images injected with agomir-53 (**A**) or antagomir-53 (**C**) after infection with *B. bassiana* conidia. Three repetitions of each experiment were conducted. The Kaplan-Meier approach was used to draw the survival curves, and the *P*-value was determined by the log-rank (Mantel-Cox) test. *P < 0.05. Protein expression level of ModSP in the *Ae. aegypti* hemolymph after agomir-53 (**B)** or antagomir-53 (**D**) treatment upon *B. bassiana* infection. LP II was used as the loading control. Agomir-C + PBS, Agomir-C + Bb, and Agomir-C-injected mosquitoes treated with PBS or *B. bassiana*; Agomir-53 + PBS, Agomir-53 + Bb, Agomir-novel-53-injected mosquitoes treated with PBS or *B. bassiana*; Antagomir-C + PBS, Antagomir-C + Bb, and Antagomir-C-injected mosquitoes treated with PBS or *B. bassiana*; Antagomir-53 + PBS, Antagomir-53 + Bb, and Antagomir-novel-53-injected mosquitoes treated with PBS or *B. bassiana*

To study the effect of miRNA-novel-53 on the antifungal immunity of *Ae. aegypti*, mortality was scored after injecting either antagomir-53 or agomir-53 into the thorax. Mosquitoes became more sensitive to fungal infection after injection of agomir-53, and the mortality was significantly greater than in adults treated with agomir-C. Mosquitoes injected with agomir-53 had greater mortality caused by *B. bassiana* (Fig. 5A). The protein level of ModSP significantly decreased in mosquitoes treated with agomir-53 compared to those treated with agomir-C (Fig. 5B). In contrast, the toxicity of *B. bassiana* to mosquitoes was weakened, and their survival was greater after treatment with antagomir-53. Mosquitoes treated with antagomir-53 were killed more slowly by the fungus (Fig. 5C). Also, the protein level of ModSP in mosquitoes treated with antagomir-53 was significantly greater than that in mosquitoes treated with antagomir-C (Fig. 5D). These results demonstrate that the miRNA-novel-53 reduces host resistance to fungal infection.

To study the influence of miRNA-novel-53 on mosquito immunity, we constructed miRNA loss-of-function (miR-53^−/−^) *Ae. aegypti* by employing CRISPR-Cas9 technology and hybrid breeding. Pairwise alignment between the wild-type and the mutant-type genome revealed that miRNA-deficient mosquitoes had a 4-bp deletion in its mature region (Additional file 2: Figure S4A). The qRT-PCR results validated that miRNA-novel-53 was knocked out in heterozygote (miR-53^+/−^) and homozygote mosquitoes (Additional file 2: Figure S4B). Conversely, the mRNA level of *ModSP* increased in the mutant hosts (Additional file 2: Figure S4C). The level of ModSP protein in the knockout mosquito hemolymph was also significantly increased (Additional file 2: Figure S4D).

**Fig. 6:**
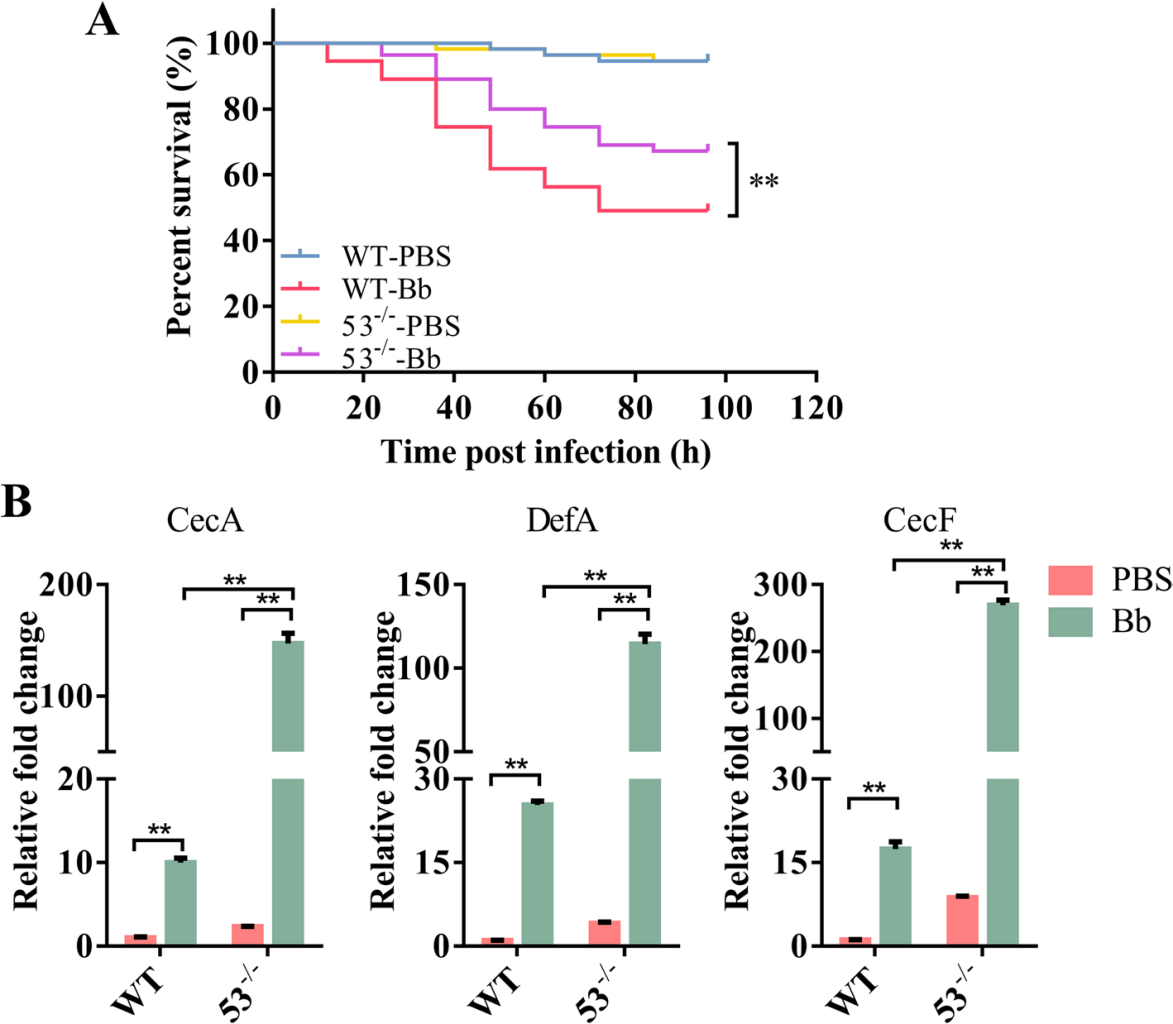
Effects of miRNA-novel-53 knockout on the antifungal immunity of *Ae. Aegypti*. **A** Survival rate of miRNA-novel-53 knockout *Ae. aegypti* after infection with *B. bassiana* conidia. Three repetitions of each experiment were conducted. The Kaplan-Meier approach was used to draw the survival curves, and the *P*-value was determined by the log-rank (Mantel-Cox) test. **P < 0.01. 53^−/−^-PBS, miRNA-novel-53 knockout mosquito treated with PBS; 53^−/−^-Bb, miRNA-novel-53 knockout mosquito infected with *B. bassiana*. **B** Expression levels of *AMPs* in the knockout mosquito line treated with PBS or *B. bassiana* conidia for 48 h. The qRT-PCR results were normalized to the internal control rps7. The results were obtained in triplicate and are presented as mean ± SEM. The Student’s *t*-test was used to determine the significance of treatment differences (GraphPad Prism 6). **P < 0.01. 53^−/−^, miRNA-novel-53 knockout mosquito

Then, the survival rate of miR-53^−/−^ mosquitoes infected with *B. bassiana* was analyzed. Compared to the wild type, their survival rate significantly increased after fungal infection (Fig. 6A). This suggests that miRNA-novel-53 is involved in antifungal immunity. Some *AMP* genes were detected in miR-53^−/−^ mosquitoes infected with *B. bassiana*. The qRT-PCR results showed that the mRNA of *AMPs* increased in both lines after *B. bassiana* infection for 48 h. Much higher mRNA levels of *AMPs* occurred in mutant mosquitoes (Fig. 6B). These results are basically consistent with those of knocking down by injection of the antagomir. The knockout experiment further supports our conclusion. Unfortunately, we were unable to obtain *ModSP* knockout mosquitoes. However, the results demonstrate that the miRNA-novel-53 reduces host defense to fungal infection.

### Novel-circ-930 functions as a miRNA-novel-53 sponge in regulating the expression of *ModSP*

To further address the role of noncoding RNA in immune responses, associated circRNAs from mosquitoes infected with *B. bassiana* were investigated (Additional file 2: Supplementary methods) [41, 42]. One of them, novel-circ-930, was detected in cDNA and genomic DNA samples from RNase R treatment by PCR. The target circRNA fragment can be amplified from cDNA samples with divergent primers, but it could not be amplified from genomic DNA samples (Additional file 2: Figure S5A). Sanger sequencing results showed that the sequence of the PCR product matched cyclization sites with reverse splicing (Additional file 2: Figure S5B, Additional file 6: Table S5). This indicated that there is a covalent closed-loop structure in the novel-circ-930 according to sequence alignment (Additional file 2: Figure S5B). These results are consistent with predictions. The expression level of novel-circ-930 significantly increased in mosquitoes infected with *B. bassiana* according to qRT-PCR (Additional file 2: Figure S5C).

**Fig. 7:**
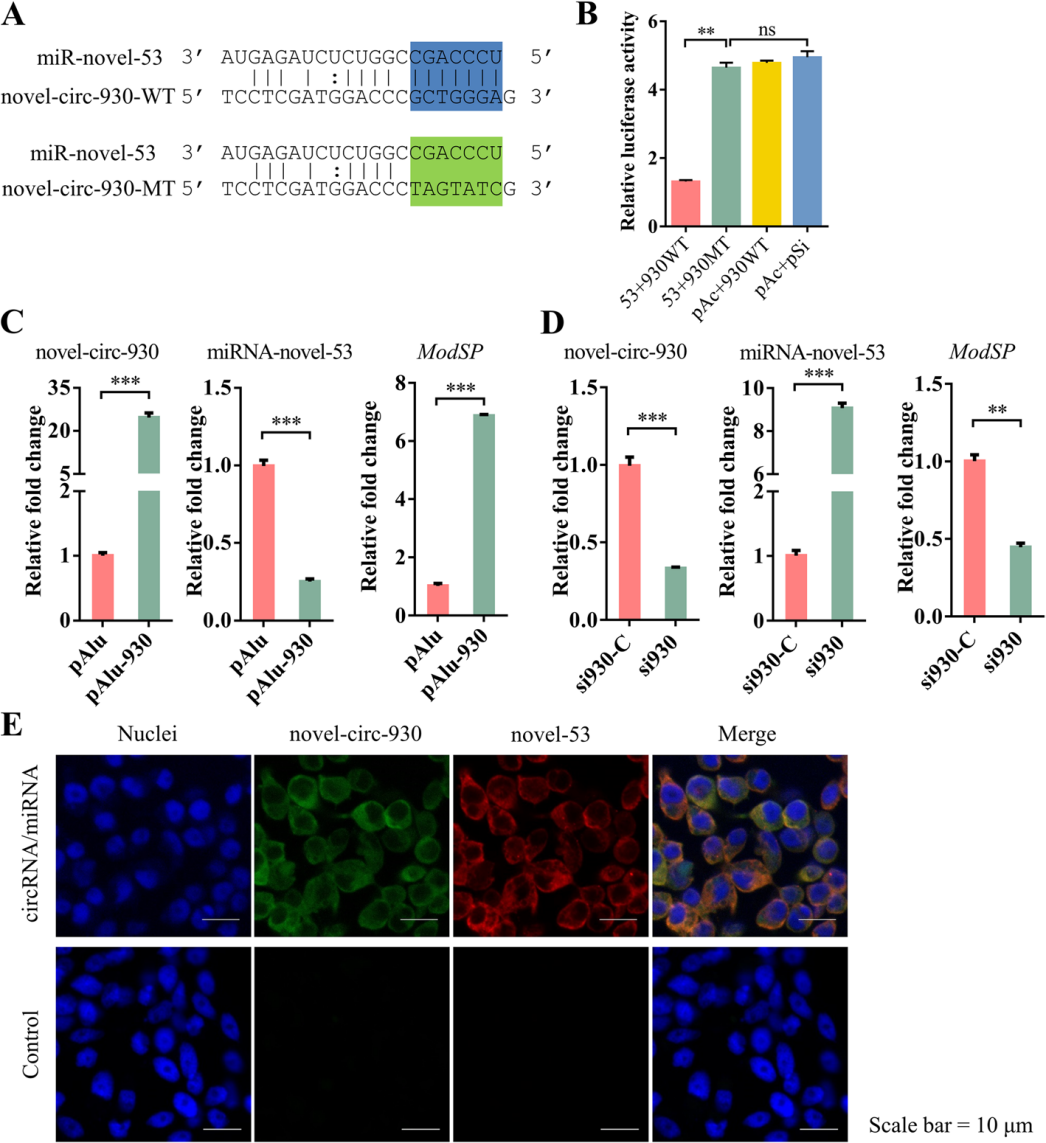
novel-circ-930 functions as a miRNA-novel-53 sponge on the antifungal immunity of *Ae. Aegypti*. **A** Sequence alignment of miRNA-novel-53 and the predicted binding site in novel-circ-930. **B** Relative luminescence activity after transfection of different plasmids in the S2 cell line. 53 + 930WT, cells transfected with pAc5.1b-miRNA-novel-53 and psiCHECK2-novel-circ-930-WT plasmids; 53 + 930MT, cells transfected with pAc5.1b-miRNA-novel-53 and psiCHECK2-novel-circ-930-MT plasmids; pAc + 930WT, cells transfected with pAc5.1b empty vector and psiCHECK2-novel-circ-930-WT plasmid; pAc + pSi, cells transfected with pAc5.1b and psiCHECK2 plasmids. Each sample had four replicates. The statistical significance of treatment differences was judged using the Student’s *t*-test. ns, *P* > 0.05; **P < 0.01. **C** Quantitative detection of overexpression of circRNA in Aag2. pAlu, cells transfected with pAlu5.1b empty vector; pAlu-930, cells transfected with pAlu5.1b-novel-circ-930. **D** Quantitative detection of interference of circRNA in Aag2. si930, cells transfected with siRNA of novel-circ-930; si930-C, cells transfected with negative siRNA of novel-circ-930. The results were obtained in triplicate and presented as mean ± SEM. The Student’s *t*-test was used to determine the statistically significant differences. **P < 0.01, ***P < 0.001. **E** Localization of miRNA-novel-53 and novel-circ-930 was performed with FISH in Aag2 cells. Green, novel-circ-930; red, miRNA-novel-53; blue, Hoechst 33342. Scale bar, 10 μm

Based on sequence alignment analysis and prediction by multiple algorithms, there is an interactive relationship between novel-circ-930 and miRNA-novel-53 (Fig. 7A). To validate this targeting relationship, we performed a double luciferase reporter experiment using recombinant vectors with target site (circ-930-WT) or mutant target site (circ-930-MT) on novel-circ-930 (Fig. 7A). The luciferase activity in S2 cells with the presence of miRNA-novel-53 and novel-circ-930 sequence decreased to nearly 30%, while the changes of activity were not obvious when the novel-circ-930 targeted site was mutated (Fig. 7B). These findings showed that miRNA-novel-53 could bind novel-circ-930.

We modified a commercial plasmid (pAc5.1b) to carry the elements necessary for the formation of a circular structure in multiple clone sites to achieve overexpression of circRNA (Additional file 2: Figure S5D). The modified plasmid was named pAlu5.1b. We found that the novel-circ-930 could successfully appear in S2 cells transfected with the plasmid inserted with the novel-circ-930 (pAlu-930) according to PCR with divergent primers (Additional file 2: Figure S5D). However, it was not amplified in samples transfected with pAlu5.1b and the plasmid inserted with the novel-circ-1041 (pAlu-1041). Also, the expression level of novel-circ-930 greatly increased in Aag2 cells transfected with vectors of novel-circ-930 insertion compared to the control plasmids through the results of qRT-PCR (Additional file 2: Figure S5D). The reliability of the overexpression vector was further verified. Then, qRT-PCR was performed to determine the abundance of miRNA-novel-53 and *ModSP* in the transfected samples. The relative abundance of miRNA-novel-53 significantly decreased, whereas the expression level of *ModSP* significantly increased in novel-circ-930 overexpressed cells (Fig. 7C). In contrast, the expression of novel-circ-930 significantly decreased in the samples transfected with its siRNA, while the expression of miRNA-novel-53 increased, and *ModSP* was downregulated (Fig. 7D). We also synthesized a probe of the circRNA to perform FISH. The novel-circ-930 was also detected to be localized in the cytoplasm, as was miRNA-novel-53 (Fig. 7E). These results indicate that mosquito novel-circ-930 can affect *ModSP* mRNA by interacting with miRNA-novel-53.

## Discussion

Serine proteases and miRNAs are major factors involved in innate immunity, but their interactions are unclear. To study how miRNA influences the expression of SPs to participate in the mosquito antifungal response, we conducted transcriptome and survival analysis. The *Ae. aegypti* ModSP was a predicted homologous protein of the MSP in *T. molitor*, the ModSP in *Drosophila*, and the HP14 in *M. sexta*. These are activated after the specific recognition of PAMPs and act as important components of their respective immune systems [9, 10, 43]. We found that ModSP responds to the *B. bassiana* infection in *Ae. aegypti*. The transcription level of *ModSP* increased to more than 2.2-fold at 48 h after infection. The protein level of ModSP had a similar change in hemolymph samples from the infected group. The mortality of infected mosquitoes was increased by *ModSP* RNAi treatment (dsModSP + Bb) compared to the control group. Knocking down ModSP in mosquitoes resulted in lower resistance to *B. bassiana* infection and reduced *AMPs* levels. This finding was consistent with that of previous research in *Ae. aegypti* [44] and *Drosophila* [9]. The reason for no significant difference of *ModSP* transcript at early stage after fungal infection might be due to the inhibition by OTU7B [45]. ModSP was recruited and achieved a level of autoactivation [9]. The increase of ModSP is a prerequisite for its activation to perform immune functions. *M. sexta* proHP14 can be activated in response to fungal infection in the presence of βGRP1 [10] or βGRP2 [11, 46]. *T. molitor* MSP is processed into the activated form by recognition of GNBP3 and β-1,3-glucan [47]. These studies on lepidopteran and coleopteran insects were conducted using biochemical methods. If in vitro reconstitution experiments could be performed, our understanding of the mosquito protease network would greatly improve.

miRNAs help regulate a variety of physiological pathways, including insect immunity [48]. Many studies have shown that miRNA plays a regulatory role as a local or systemic factor in insect immune system. In *Anopheles gambiae*, miRNA-305 helps control the anti-*Plasmodium* response and midgut microbiota, most likely by posttranscriptional alteration of immune effector genes [49]. *Plutella xylostella* miR-8 modulates Serpin 27 at the transcription level. Serpin 27 is a serine protease inhibitor that influences the Toll pathway and melanization reaction [50]. Interestingly, *Drosophila* with miR-317 transiently overexpressed had a higher mortality. However, miR-317 knockout *Drosophila* had lower mortality than the control group after gram-positive bacterial infection. This revealed miRNA involvement in the interaction between *Drosophila* immunity and survival [27]. miRNAs derived from pathogens can also act on the host. *B. bassiana* deploys a cross-kingdom microRNA-like RNA that inhibits mosquito immunity by decreasing the expression of Spätzle 4 and facilitating infection [51].

In this study, we found that miRNA-novel-53 regulates the levels of ModSP basally expressed in *Ae. aegypti*. After fungal infection, the expression of miRNA-novel-53 decreased, while that of ModSP significantly increased. Unlike the interaction regions between most miRNAs and their target genes, the *Ae. aegypti ModSP* binding sequence of miRNA-novel-53 occurs in the CDS, not the 3′ UTR or 5′ UTR as seen previously [52–54]. Although the majority of discovered miRNAs were found to bind to the UTR of target genes, new evidence suggests that some miRNAs can bind to the CDS of target genes in animals [55–57]. This domain-dependent interaction may contribute to the diversity of posttranscriptional gene expression regulation [57]. FISH was performed in the Aag2 cell line to determine where miRNA-novel-53 may act on *ModSP*. The fluorescence results indicated that miRNA-novel-53 and *ModSP* are both localized in the cytoplasm. These results are similar to those of previous studies showing that miRNA and its target gene both play roles in the cytoplasm [58, 59]. Mature miRNA-novel-53 is transported into the cytoplasm to inhibit the translation of *ModSP* transcripts and downregulate its expression level. *Drosophila melanogaster* C virus (DCV) infection resulted in a reduction of miR-8-5p or miR-956. Their targeted genes were negatively regulated in viral infection [60, 61].

Chemical pesticides can pollute the environment and harm nontarget organisms. *B. bassiana* is a useful biopesticide that has ecological benefits. However, there is a risk that target pests will evolve reduced susceptibility or resistance to fungi [62, 63]. Therefore, enhancing the pathogenicity of the pathogen or weakening the immunity of the pest insect host could be useful resistance management strategies. The survival of infected mosquitoes decreased significantly after injecting the dsRNA of *ModSP* to interfere with its expression. Intrathoracic inoculation of the miRNA-novel-53 agomir and antagomir can regulate the transcription level and protein level of the target gene, thus interfering with the immune activation of mosquitoes. miRNA loss-of-function (miR-53^−/−^) *Ae. aegypti* was obtained by employing CRISPR-Cas9 technology. The mRNA level of *ModSP* increased in the mutant mosquitoes, and their survival significantly increased after fungal infection compared to wild type. Much higher mRNA levels of *AMPs* were found in mutant mosquitoes. These results are consistent with those resulting from knocking down by injecting antagomir. The knockout experiment further supports our conclusion. These findings showed that miRNA-novel-53 might have potential as a new effector in the development of improved biopesticides.

Many mechanisms of immunological inhibition have been discovered. The Toll pathway and activation of PPO3 in the hemolymph of mosquitoes infected with *B. bassiana* can be repressed by CLSP2 interacting with the recognition molecule TEP22 [44]. *Drosophila* miR-959–962 cluster can negatively regulate the Toll pathway by binding to the 3′ UTR of *tube* or *Toll* mRNAs to protect normal systems from overactivation of the immune reaction and achieve immune homeostasis during the late phase of infection [64]. As a vital immune initiating molecule, ModSP needs to be suppressed in normal organism to avoid the detriment caused by subsequent immune factors. In physiological condition, miRNA-novel-53 can inhibit the translation of ModSP by targeting its CDS region, which maintains a basic level of ModSP expression. The reduction of miRNA-novel-53 eliminated the repression to *ModSP* mRNA. The translation level of ModSP significantly increases when defending against fungal infection.

Other noncoding RNAs derived from the host or pathogen, including long noncoding RNAs (lncRNAs) and circRNAs, typically build a functional network with miRNAs to modulate target mRNAs. These noncoding RNAs might cause a decrease in miRNA after immune challenge. One competing endogenous RNA (ceRNA) regulatory system with three lncRNAs, two miRNAs, and nine mRNAs was constructed in diabetic cardiovascular diseases [65]. Another study identified a regulatory mechanism in which lncRNA-CR11538 interacts with transcription factors Dif/dorsal to suppress antimicrobial peptide expression and restore *Drosophila* Toll immune homeostasis [66]. A constructed circRNA/miRNA/mRNA network showed that silkworm circRNAs can act as miRNA sponges to participate in the immune response against BmNPV by genome-wide RNA-Seq [35]. Similarly, some predicted circRNAs could cause reduction of miRNA-novel-53 after *B. bassiana* infection. Here, we identified a novel-circ-930/miRNA-novel-53/*ModSP* axis that functions as a regulator to modulate antifungal immunity in mosquito.

## Conclusions

**Fig. 8.**
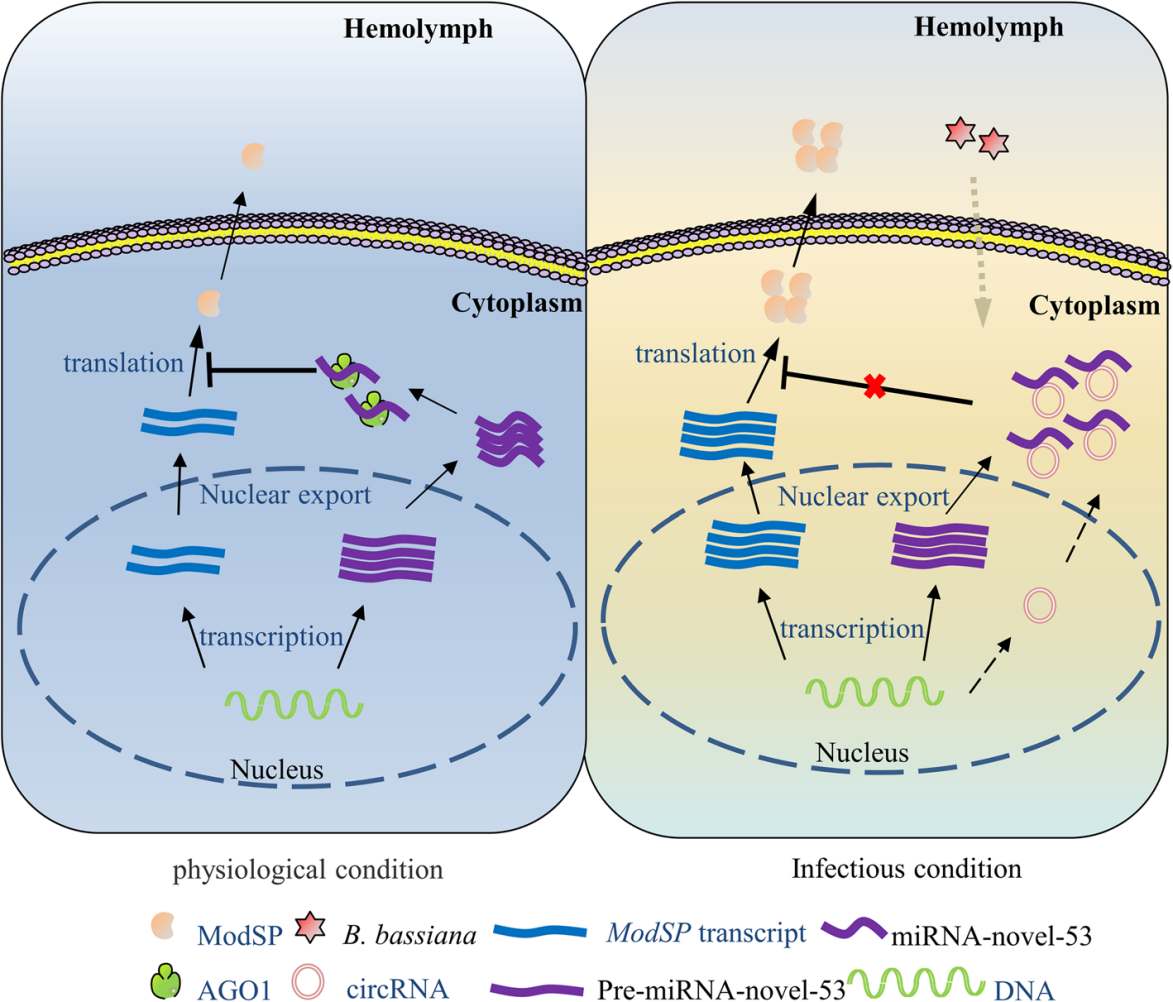
Schematic diagram of the novel-circ-930, miRNA-novel-53, and ModSP response to fungal infection. Left panel: physiological condition, miRNA-novel-53 with silencing complex inhibits the translation of ModSP by targeting its CDS region in the cytoplasm, which maintains the basic expression level of ModSP. Right panel: infectious condition, the reduction of miRNA-novel-53 sponged by novel-circ-930 leads to an elimination of the repression of *ModSP* mRNA. The protein level of ModSP then significantly increases to defend fungal infection

The expression level of ModSP significantly increased in *Ae. aegypti* after infection with *B. bassiana*. Mosquitoes injected with dsModSP were more sensitive to *B. bassiana* than those treated with dsEGFP. This study identified a new miRNA, miRNA-novel-53, which was greatly reduced after infection. Dual luciferase reporter experiment showed that the new miRNA could diminish *ModSP* expression by directly interacting with its CDS region. FISH demonstrated that the cytoplasm is the location in which mature miRNA-novel-53 affects the expression of the target mRNA. Overexpression of miRNA-novel-53 reduced ModSP at both mRNA and protein levels. The mortality of miRNA-overexpressing mosquitoes also increased after fungal infection. In contrast, a contrary result occurred after miRNA knockout or antagomir injection. The novel-circ-930 act as a miRNA-novel-53 sponge to modulate target genes according to transfection with siRNA or overexpression plasmid. These findings indicate that novel-circ-930 affects the expression level of ModSP by a novel-circ-930/miRNA-novel-53/*ModSP* axis (Fig. 8), which functions as a regulator in the host immune system.

## Methods

### Feeding and immune infection

*Aedes aegypti* mosquitoes (Liverpool strain) were used for the experiments. The feeding conditions were identical to the previous programs in our laboratory [37, 67]. Adult mosquitoes were continuously provided with water and a 10% (wt/vol) sucrose solution. Mosquitoes were fed with chicken blood to start the reproduction process. Immune challenge by *B. bassiana* (ARSEF2860) was performed as described previously [44]. All procedures involving the use of experimental animals were approved by the Animal Care and Use Committees of Institute of Zoology, Chinese Academy of Sciences (Beijing, China).

### RNA-sequencing

Six individual mosquitoes for each sample were smashed and lysed with TRIzol reagent (Invitrogen, Carlsbad, CA, USA). High-quality total RNA from mosquitoes treated with PBS or *B. bassiana* for 48 h was achieved according to the manufacturer’s instructions. Three biological replicates were established in each group. RNA-Seq libraries were constructed following the information in the Ultra™ Directional RNA Library Prep Kit (Illumina, NEB, USA). High-quality libraries were validated using an Agilent Bioanalyzer 2100 system (Agilent Technologies, USA). Then, sequencing was performed on Illumina HiSeq 4000 system (Novogene, China).

High-quality clean data were obtained from raw reads via a validated Perl algorithm, which filtered adapter-containing reads, poly-N containing reads, and low-quality reads. Subsequent data computation was performed using these high-quality clean data. The files of the *Ae. aegypti* reference genome and gene model annotation were downloaded from VectorBase (https://vectorbase.org/vectorbase/app/record/organism/TMPTX_aaegLVP_AGWG) directly. A reference genome index was created by running Bowtie v2.2.8 [68], which was matched against the clean data.

### Differential expression analysis

Transcripts per million (TPM) was used to calculate the quantity of miRNA or mRNA expression using the basic guidelines [69]. Normalized expression = matched read count/total reads × 1,000,000. Then, DESeq2 [70] was chosen to execute differential expression analysis of the different conditions. Screening conditions for significant differential expression were adjusted *P*-value < 0.05 by default.

### miRNA alignment and target gene prediction

Matched small RNA tags were selected to search for discovered miRNA. The miRBase Release 20.0 was chosen as the existing data pool. Evolved algorithm code miRDeep2 [71] was executed to acquire the presumable miRNAs and simulate their secondary structure. miREvo [72] and miRDeep2 [71] were used to obtain novel miRNAs based on the hairpin structure features of their miRNA precursor.

The seed sequence was highly conserved and was found at sites 2–8 of a mature miRNA. With the alteration of nucleotides in this short region, the target of a miRNA might change. In the algorithm analysis, miRNA that may have base edition capacity could be found by matching all small RNA tags to mature miRNA and permitting single-base mispairing. Determining the target gene of a miRNA in this study was fulfilled with miRanda [73].

### Quantification of transcription level

Each sample of total RNA was prepared from 6 to 8 mosquitoes with TRIzol (Invitrogen, Carlsbad, CA, USA). For mRNA transcription, cDNA was generated from total RNA with the RT PreMix Kit (Accurate Biology, China). For circRNA transcription, cDNA was synthesized from the RNase R-treated (Epicentre, USA) total RNA samples with the RT Premix kit. For miRNA transcription, the Takara miRNA First-Strand Synthesis Kit (Takara, Japan) was used to produce cDNA. Then, quantitative real-time PCR (qRT-PCR) was performed with SuperReal PreMix Plus (Tiangen, China) for the cDNA of mRNA or miRNA on StepOnePlus instrument (Thermo Fisher, USA). Additional file 7: Table S6 lists all of the qRT-PCR primers. The 2^−ΔΔCt^ calculated approach of relative quantification was used to count the qRT-PCR results, with the internal controls rps7 for mRNA and circRNA and U6 for miRNA. The results are presented in the form of mean ± SEM, and analysis of statistical difference analysis between groups was assessed with the Student’s *t*-test (GraphPad Prism 6.0).

### RNA interference

PCR was used to produce 500–900-bp cDNA samples of target genes with primers linked to T7-phage promoter regions. T7 RNA polymerase was added to synthesize dsRNA by simultaneous transcription of both strands of the purified cDNA samples. Control-enhanced green fluorescent protein (EGFP) dsRNA was produced using the EGFP gene. Microinjection was accomplished using a nanoliter 2000 injector (World Precision Instruments, USA). Corresponding dsRNA was injected into the thorax of cold-anesthetized female mosquitoes within 1 day after their emergence. Additional file 7: Table S6 lists all of the qRT-PCR primers.

### Dual luciferase reporter assays

The fragment centered on miRNA-novel-53 precursors on the genome was cloned into vector pAc5.1/V5-HisB (Invitrogen, USA). The correct fragment around predicted target sites in *ModSP* or novel-circ-930 was integrated into vector psiCHECK-2 (Promega, USA). The predicted target regions of them were replaced by the overlap extension PCR. The psiCHECK-2 vectors inserted into target segments (WT or MT) were co-transfected with the miRNA expression vector into *Drosophila* S2 cells by Lipofectamine 3000 (Thermo Fisher Scientific, USA). Then, luciferase activity was detected at 40-h post transfection with a GloMax 96 Microplate Luminometer (Turner Biosystems, USA). Additional file 7: Table S6 lists all of the qRT-PCR primers.

### miRNA agomir/antagomir injection

Synthesized miRNA agomir or antagomir was used to study the endogenous miRNA abundance. The miRNA-novel-53 agomir (50 pM) (agomir-53) or antagomir (50 pM) (antagomir-53) (Shanghai GenePharma Co., Ltd.) was introduced into anesthetized female mosquitoes within 1-day post-eclosion. Control mosquitoes were introduced with the cel-miR-67-3p agomir (5′-UCACAACCUCCUAGAAAGAGUAGA-3′) (50 pM) (agomir-C) or antagomir (50 pM) (antagomir-C). The miRNA expression level after injection was confirmed by qRT-PCR. The injected mosquitoes recovered for 3 days before subsequent infection. In addition, qRT-PCR was used to detect the expression of specific genes.

### Generation of polyclonal antibody

Full length of ModSP was cloned into the prokaryotic expression vector pET-28a. The recombinant plasmids were transformed into *Escherichia coli* strain BL21 (DE3). Recombinant ModSP protein was expressed and purified by Ni^2+^ affinity chromatography (QIAGEN, Germany) from the BL21 cells. The protein was then used as the antigen for generation of polyclonal antibodies by Beijing Protein Innovation.

### Immunoblot analysis

Hemolymph samples were acquired from 20 beheaded mosquitoes with a QIAshredder column (QIAGEN, Germany) containing a protease inhibitor by centrifugation at 4000 g for 5 min [40]. Total protein samples were extracted by using RIPA lysis buffer (CWBIO, China). The protein samples were run on a 4–20% gradient SDS-PAGE (RSBM, China) and transferred to a polyvinylidene difluoride membrane (Millipore, Germany). The blocked membrane was immersed with specific antibody (1:5000) overnight at 4 °C. *Aedes aegypti* lipophorin II (LP II) antibody was used as the loading control in different samples [40, 44]. The corresponding secondary antibody (1:10,000) was incubated at room temperature for 100 min. SuperSignal PLUS (Thermo Scientific, USA) was utilized to observe immunological blots.

### Synthesis of probe and FISH

miRNA and circRNA probes were designed and synthetized at Shanghai GenePharma Co., Ltd. mRNA probes were prepared using an NTP labeling mixture and an SP6/T7 Transcription Kit (Roche, Basil, Switzerland). They were then broken into small fragments of about 250 bp by alkaline lysis. RNA fluorescence in situ hybridization (FISH) was carried out as per the manufacturer’s instructions and previous methods [74], with a FISH kit (Guangzhou RiboBio Co., Ltd.). Briefly, the adherent cells were prepared with 4% PFA and immersed in PBS containing 0.3% Triton X-100. The samples were hybridized with miR-novel-53 probe (10 pM) and *ModSP* probe (20 ng/μL) or circRNA probes (20 pM) at 37 °C approximately 12 h after treatment with proteinase K (25 g/mL) at 37 °C for 20 min and pre-hybridization for 5 h. Then, they were rinsed, in order, in 2 × SSC, 1 × SSC, and 0.2 × SSC at 37 °C. Mouse anti-digoxin antibody (1:150) and rabbit anti-biotin antibody (1:150) were used at 4 °C approximately 12 h for probe detection. The cells were then incubated with FITC-conjugated goat anti-rabbit and Alexa Fluor 594-conjugated goat anti-mouse (Bioss Antibodies, China) for 60 min in darkness. HNPP/Fast Red (Roche, Basil, Switzerland) or fluorescein-tyramide (Perkin Elmer, USA) was used to acquire the fluorescent signal. All images were captured with a LSM 710 (Carl Zeiss AG, Germany).

### RNA immunoprecipitation (RIP)

An RIP Kit (Novogene, China) was used to carry out the assay with minor modifications. The RIP lysis buffer in the kit was used to homogenize the samples from different groups. The upper liquid was then mixed at 4 °C overnight with beads preincubated with either an antibody against *Ae. aegypti* AGO1 or a commercial antibody of IgG following high-speed freeze centrifugation. After RNA extraction from the protein-miRNA complexes, reverse transcription was performed for the enriched miRNA. Then, qRT-PCR was performed to quantify the expression of miRNA.

### Generation of miRNA mutant mosquito

Prediction of sgRNA sequences was performed online (http://crispor.tefor.net/) according to the DNA sequences of selected miRNA segments. The sgRNAs were synthesized by using a T7 Express RNAi kit (Promega, USA). The mixture of Cas9 protein (300 ng/μL) (PNA Bio, USA) and sgRNAs (50 ng/μL) was injected into freshly laid embryos using a micromanipulator (Eppendorf, Germany). The injected embryos were hatched at 6 days after treatment. Genotypes of all adult mosquitoes were identified by Sanger sequencing. Chimeric adult mosquitoes were individually mated with wild-type (WT) mosquitoes. Their offspring (G1) were cultured to adulthood under standard conditions. Heterozygote mosquitoes were obtained by sequencing and then hybridized. The mutation in this knockout genotype is heritable. Heterozygote mosquitoes (G2) with the same genotype were selected to mate with WT mosquitoes. Homozygous mosquitoes were identified from the offspring by heterozygous selfing at G3. The miRNA knockout *Ae. aegypti* was constructed successfully by homozygous selfing for three generations.

### CircRNA overexpression or suppression

Synthesized siRNA of circRNA (Shanghai GenePharma Co., Ltd.) or overexpression vector was used to study the endogenous circRNA abundance. The novel-circ-930 siRNA (100 nM) (si930) (Shanghai GenePharma Co., Ltd.) or control siRNA (100 nM) (si930-C) was transfected into Aag2 cells. The novel-circ-930 overexpression plasmid (pAlu-930) or control plasmid (pAlu) was transfected into Aag2 cells or S2 cells. The expression level of circRNA after transfection was detected by PCR and qRT-PCR. In addition, qRT-PCR was used to detect the expression of specific genes.

### Survival rate analysis

Fungal challenge was performed with *B. bassiana* conidia (1.0 × 10^8^ /mL) by the needle pricking method after treatment with dsRNA, agomir, or antagomir for 72 h. A total of 55 female mosquitoes, within 3 days after emergence, were used in each group. The treated mosquitoes were maintained under normal conditions for 4 days. Each experiment was carried out with at least three independent repetitions. The obtained data were processed and displayed using GraphPad Prism 6.0 software. The Kaplan–Meier approach was used to generate survival curves, and the *P*-value was determined by the log-rank (Mantel-Cox) test.

### Supplementary Information

**Additional file 1:** **Table S1.** Analysis of differentially expressed mRNAs response to *B. bassiana* infection in *Ae. aegypti*.

**Additional file 2:** **Supplementary methods and figures.** Supplementary methods. **Figure S1.** Phylogenetic analysis of modular SPs. (A) The signs in the figures indicate ModSP. The amino acid sequences of *H. armigera* (Ha), *D. melanogastor* (Dm), *Ae. aegypti* (Aa), *M. sexta* (Ms), and *An. gambiae* (Ag) SPs were aligned and constructed by MEGA 7.0 and Evolview. (B) Expression analysis of multiple SPs 48 hours after fungal infection. (C) Expression analysis of *ModSP* at 6 and 12 hours after fungal infection. (D) The RNAi efficiency was validated after dsRNA injection by qRT-PCR. **Figure S2.** Identification of miRNA-novel-53 precursor and mature form. (A) Sequence confirmation of miRNA-novel-53 precursor. (B) Hairpin structure prediction of miRNA-novel-53 precursor. Predicted by RNAstructure 6.3 software. ΔG = -25.60 kcal/mol. (C) Northern blot to detect the size of miRNA to characterize the authenticity of novel miRNA in different samples. **Figure S3.** Temporal-spatial expression of *ModSP* and miRNA-novel-53 in *Ae. aegypti*. The relative mRNA abundance of *ModSP* (A) and miRNA-novel-53 abundance (B) at different stages (DS) of larvae of adult *Ae. aegypti*. The relative mRNA abundance of three *AMPs* (C, D, and E) at different stages. The relative expression of *ModSP* (F) and miRNA-novel-53 (G) at different tissues of adult mosquitoes. The results were performed three times and displayed in the form of mean ± SEM, and statistical difference analysis between samples was assessed using the Student’s t-test. * *P* < 0.05, ** *P* < 0.01. dph, days post-hatching. HD, head. FB, fat body. MG, midgut. MT, Malpighian tubules. OV, ovary. The control group in Figure A, C, E, F, and G is mosquito larvae of 1 days post-hatching. **Figure S4.** Verification of miRNA-novel-53 in *Ae. aegypti*. (A) Pairwise alignment between wild-type and the mutant-type at genome. (B) Relative quantification of miRNA in heterozygote and homozygote mosquitoes. (C) Relative quantification of *ModSP* mRNA in heterozygote and homozygote mosquitoes. (D) The abundance of ModSP protein in the knockout mosquito hemolymph. WT, wild-type mosquito; 53^+/-^, heterozygote of miRNA-novel-53 knockout; 53^-/-^, homozygote of miRNA-novel-53 knockout. **Figure S5.** Validation and overexpression of novel-circ-930 in *Ae. aegypti*. (A) Qualitative validation of predicted circRNAs. (B) Sanger sequencing result of the validated circRNA. (C) Relative quantification of circRNAs after *B. bassiana* infection. (D) Assembly and verification of circRNA overexpression vector. (N = A, T, C, and G; R = A or C; Y = U or C). pAlu, cells transfected with pAlu5.1b empty vector; pAlu-930, transfected with pAlu5.1b-novel-circ-930 cells; pAlu-1041, transfected with pAlu5.1b-novel-circ-1041 cells. pAlu and pAlu-1041 were used as negative controls.

**Additional file 3:** **Table S2.** The amino acid sequences of SPs/SPHs in several insects.

**Additional file 4:** **Table S3.** Precursor sequence and mature sequence of known and novel miRNAs.

**Additional file 5:** **Table S4.** Analysis of differentially expressed miRNAs response to *B. bassiana* infection in *Ae. aegypti*.

**Additional file 6:** **Table S5.** Sanger sequencing results and genome sequence of the novel-circ-930.

**Additional file 7:** **Table S6.** Primers used for qRT-PCR, RNAi, probe synthesis, and gene cloning.

**Additional file 8.** Additional file containing raw data generated in this study.

## Data Availability

The BioProject accession numbers of high-throughput mRNA and small RNA sequencing data were PRJNA871933 and PRJNA871939, respectively. The sequencing reads have been stored in the NCBI SRA database according to accession numbers (mRNA from mosquitoes treated with PBS: SRR21154504, SRR21154503, SRR21154502; mRNA from mosquitoes treated with *B. bassiana*: SRR21154501, SRR21154500, SRR21154499; miRNA from mosquitoes treated with PBS: SRR21155490, SRR21155489, SRR21155488; miRNA from mosquitoes treated with *B. bassiana*: SRR21155487, SRR21155486, SRR21155485). Additionally, the accession number 31253.11.sciencedb.02643 for these data is assigned by the Science Data Bank. We confirm that all the supporting data analyzed and regarding material availability in this research are included in the article and its appendix (Additional file 8).

## Abbreviations

ModSP: Modular serine protease
FISH: Fluorescence in situ hybridization
EGFP: Enhanced green fluorescent protein
UTR: Untranslated region
RNAi: RNA interference
CDS: Coding sequence
dsRNA: Double-stranded RNA
PBS: Phosphate-buffered saline pH 7.4
