# Supplementary material for: The accumulation of modular serine protease mediated by a novel circRNA sponging miRNA increases Aedes aegypti immunity to fungus
Source: BMC Biol. 2024 Jan 17;22:7. doi: 10.1186/s12915-024-01811-6 (PMC10795361; doi:10.1186/s12915-024-01811-6)
Supplement: Supplementary file 2 — Additional file 2: Supplementary methods and figures. Supplementary methods. Figure S1. Phylogenetic analysis of modular SPs. (A) The signs in the figures indicate ModSP. The amino acid sequences of H. armigera (Ha), D. melanogastor (Dm), Ae. aegypti (Aa), M. sexta (Ms), and An. gambiae (Ag) SPs were aligned and constructed by MEGA 7.0 and Evolview. (B) Expression analysis of multiple SPs 48 hours after fungal infection. (C) Expression analysis of ModSP at 6 and 12 hours after fungal infection. (D) The RNAi efficiency was validated after dsRNA injection by qRT-PCR. Figure S2. Identification of miRNA-novel-53 precursor and mature form. (A) Sequence confirmation of miRNA-novel-53 precursor. (B) Hairpin structure prediction of miRNA-novel-53 precursor. Predicted by RNAstructure 6.3 software. ΔG = -25.60 kcal/mol. (C) Northern blot to detect the size of miRNA to characterize the authenticity of novel miRNA in different samples. Figure S3. Temporal-spatial expression of ModSP and miRNA-novel-53 in Ae. aegypti. The relative mRNA abundance of ModSP (A) and miRNA-novel-53 abundance (B) at different stages (DS) of larvae of adult Ae. aegypti. The relative mRNA abundance of three AMPs (C, D, and E) at different stages. The relative expression of ModSP (F) and miRNA-novel-53 (G) at different tissues of adult mosquitoes. The results were performed three times and displayed in the form of mean ± SEM, and statistical difference analysis between samples was assessed using the Student’s t-test. * P < 0.05, ** P < 0.01. dph, days post-hatching. HD, head. FB, fat body. MG, midgut. MT, Malpighian tubules. OV, ovary. The control group in Figure A, C, E, F, and G is mosquito larvae of 1 days post-hatching. Figure S4. Verification of miRNA-novel-53 in Ae. aegypti. (A) Pairwise alignment between wild-type and the mutant-type at genome. (B) Relative quantification of miRNA in heterozygote and homozygote mosquitoes. (C) Relative quantification of ModSP mRNA in heterozygote and homozygote mo [file 12915_2024_1811_MOESM2_ESM.docx]

## Supplementary methods

## Northern blot

More than 60 μg of total RNA was extracted with TRIzol for sRNA analysis by a Northern blot kit (BersinBio, China). Briefly, RNA samples were separated on a denaturing urea polyacrylamide gel. After the electrophoresis, RNAs were capillary transferred, for 11 h, to a piece of Hybond NX membrane (Amersham, UK). Then, the membrane was exposed to ultraviolet light to fix the RNA to the blot. The cross-linked membrane was hybridized at 42 ℃ overnight with a prepared digoxin-labeled probe (Shanghai ZhanBiao, China). The U6 probe with digoxin was used as the internal control. Table S1 lists the sequences of probes used. The membrane was incubated with anti-digoxin-AP after strict washes. The amplified signals were detected with an X-ray film.

## CircRNA sequencing

6 individual mosquitoes were smashed and lysed by TRIzol reagent (Invitrogen, Carlsbad, CA, USA). A total amount of 5 μg RNA each sample was prepared. In the beginning, the sample was treated by Ribo-zero™ rRNA Removal Kit (Epicentre, USA), and rRNA-depleted residue was cleaned up by ethanol precipitation. Then RNase R (Epicentre, USA) was added into the rRNA-free RNA sample in order to remove linear RNAs and enrich pure circRNAs. The spectrophotometer DS-11 FX+ (DeNovix Inc, USA) was used to assess the quantity and quality of it in the process. Three biological replicates were set in each group. RNA-seq libraries were constructed following the instructions of NEBNext^®^ Ultra™ Directional RNA Library Prep Kit (Illumina, NEB, USA). The quality of libraries was assessed on the Agilent Bioanalyzer 2100 system. Then, sequencing was performed on an Illumina Hiseq 4000 platform (Illumina, USA) at the Novogene Bioinformatics Technology Co., Ltd (Beijing, China). 150 bp paired-end reads were got in the process.

## CircRNA identification

Many circRNAs were systematically predicted and identified by find_circ and CIRI2. According to the sequence alignment results of bwa, PCC (paired chiastic clipping) signal, PEM (pair end mapping) signal and GT-AG splicing site information were searched by CIRI2, which could preliminarily determine junction reads. Then, the candidate circRNAs were filtered according to the results of dynamic programming alignment and genomic annotation information. Finally, the length and expression level of circRNAs were adjusted according to the results of CIRI2 identification and bwa alignment results.

## CircRNA-miRNA interaction network analysis

miRNA predicted binding sites of circRNAs were analysed using miRanda and TargetScan software base on the alignment against the microRNA database (<http://www.mirbase.org/>). According to the potential interaction by conserved seed-matching sequence between miRNAs and circRNAs, cytoscape software 3.6.1 was used to draw the circRNA-miRNA network diagrams for visualizing.

## PCR validation of circRNA

Genomic DNA was obtained from the mosquitoes using Universal Genomic DNA Kit (Zomanbio, Beijing, China). Genomic DNA was considered as a negative control for divergent primers to amplify potential circRNAs. The extracted total RNA by TRIzol reagent was incubated with RNase R to remove the linear RNA. For circRNA transcription, cDNA was synthesized from the RNase R-treated RNA sample with the RT Premix kit (Accurate Biology, China). Divergent primers were designed to validate the predicted circRNAs by PCR.

## Supplementary figures


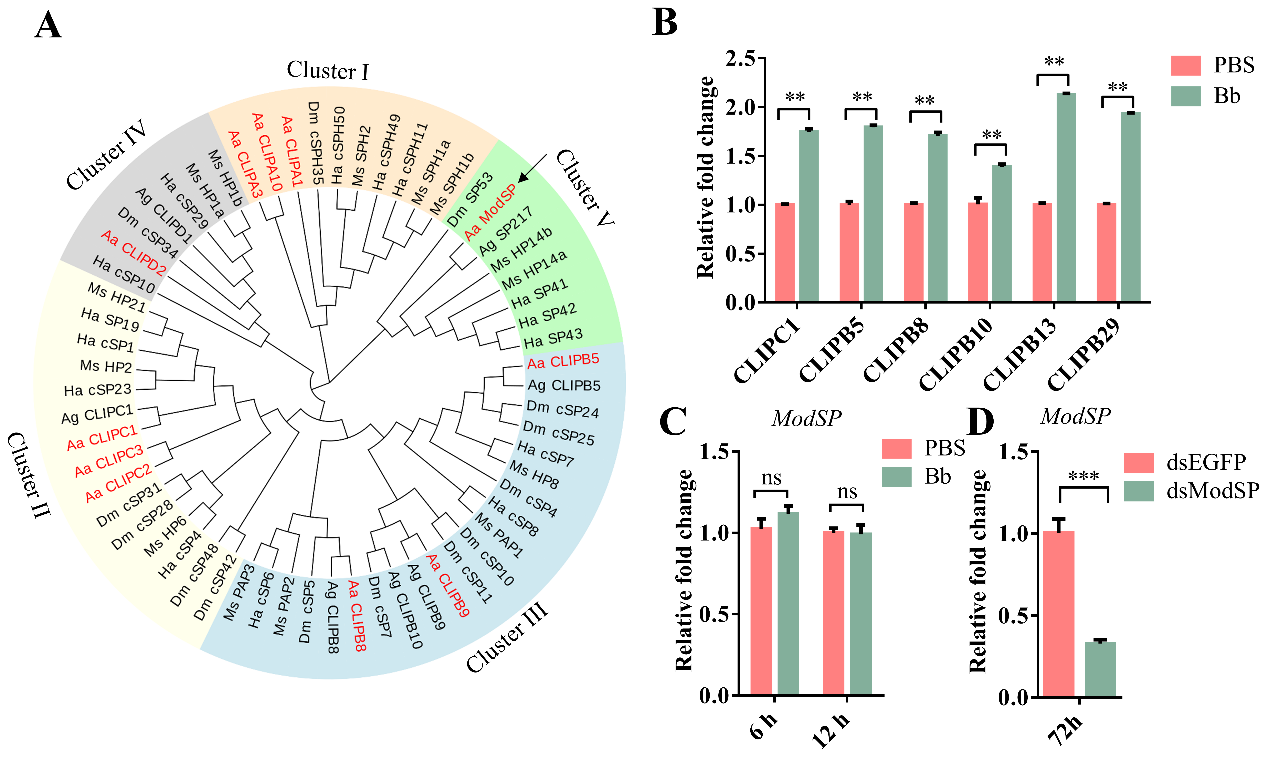


**Figure S1.** Phylogenetic analysis of modular SPs. (A) The signs in the figures indicate *ModSP*. The amino acid sequences of *H. armigera* (Ha), *D. melanogastor* (Dm), *Ae. aegypti* (Aa), *M. sexta* (Ms), and *An. gambiae* (Ag) SPs were aligned and constructed by MEGA 7.0 and Evolview. (B) Expression analysis of multiple SPs 48 hours after fungal infection. (C) Expression analysis of *ModSP* at 6 and 12 hours after fungal infection. (D) The RNAi efficiency was validated after dsRNA injection by qRT-PCR.


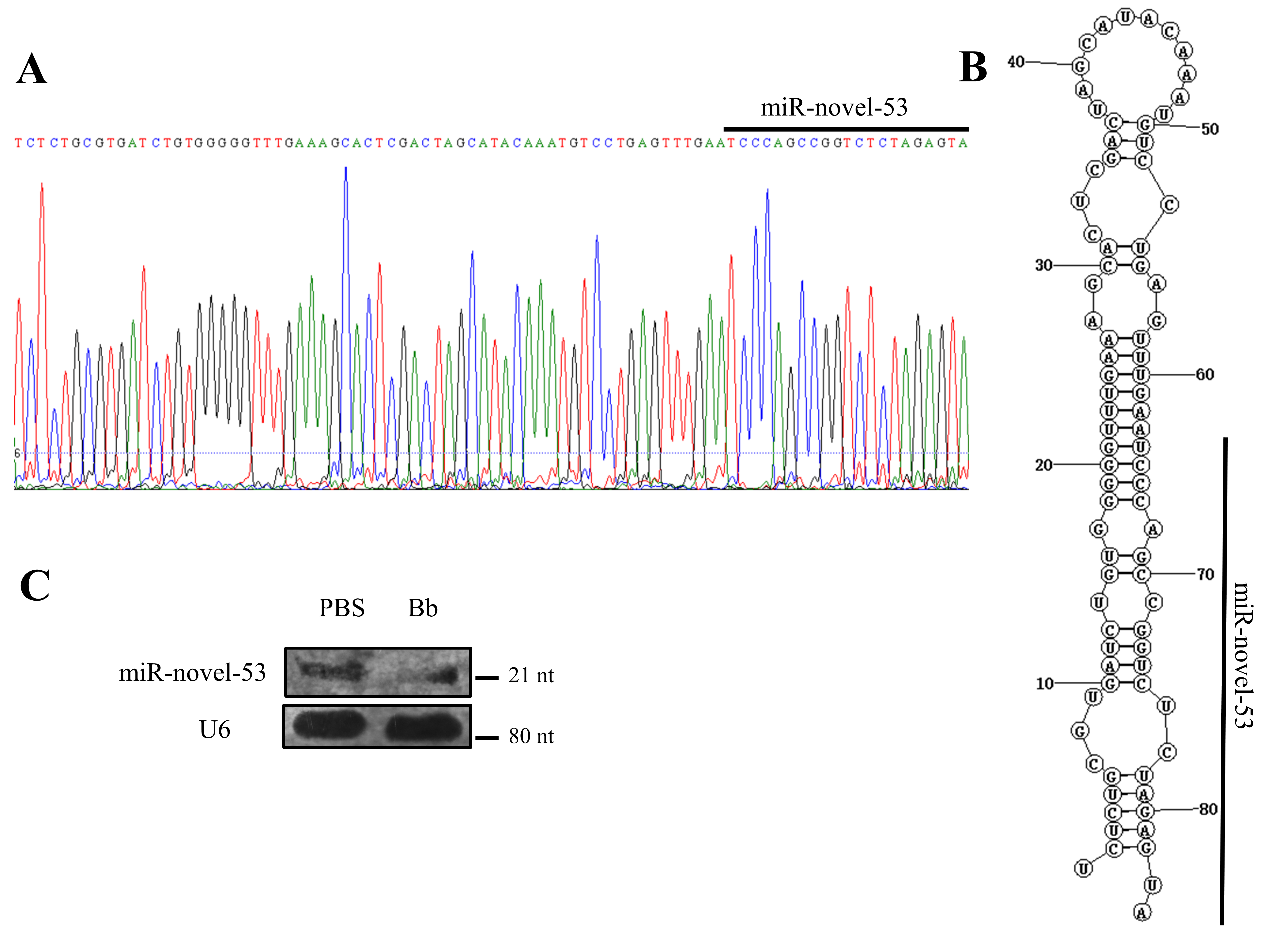


**Figure S2.** Identification of miRNA-novel-53 precursor and mature form. (A) Sequence confirmation of miRNA-novel-53 precursor. (B) Hairpin structure prediction of miRNA-novel-53 precursor. Predicted by RNAstructure 6.3 software. ΔG = -25.60 kcal/mol. (C) Northern blot to detect the size of miRNA to characterize the authenticity of novel miRNA in different samples.


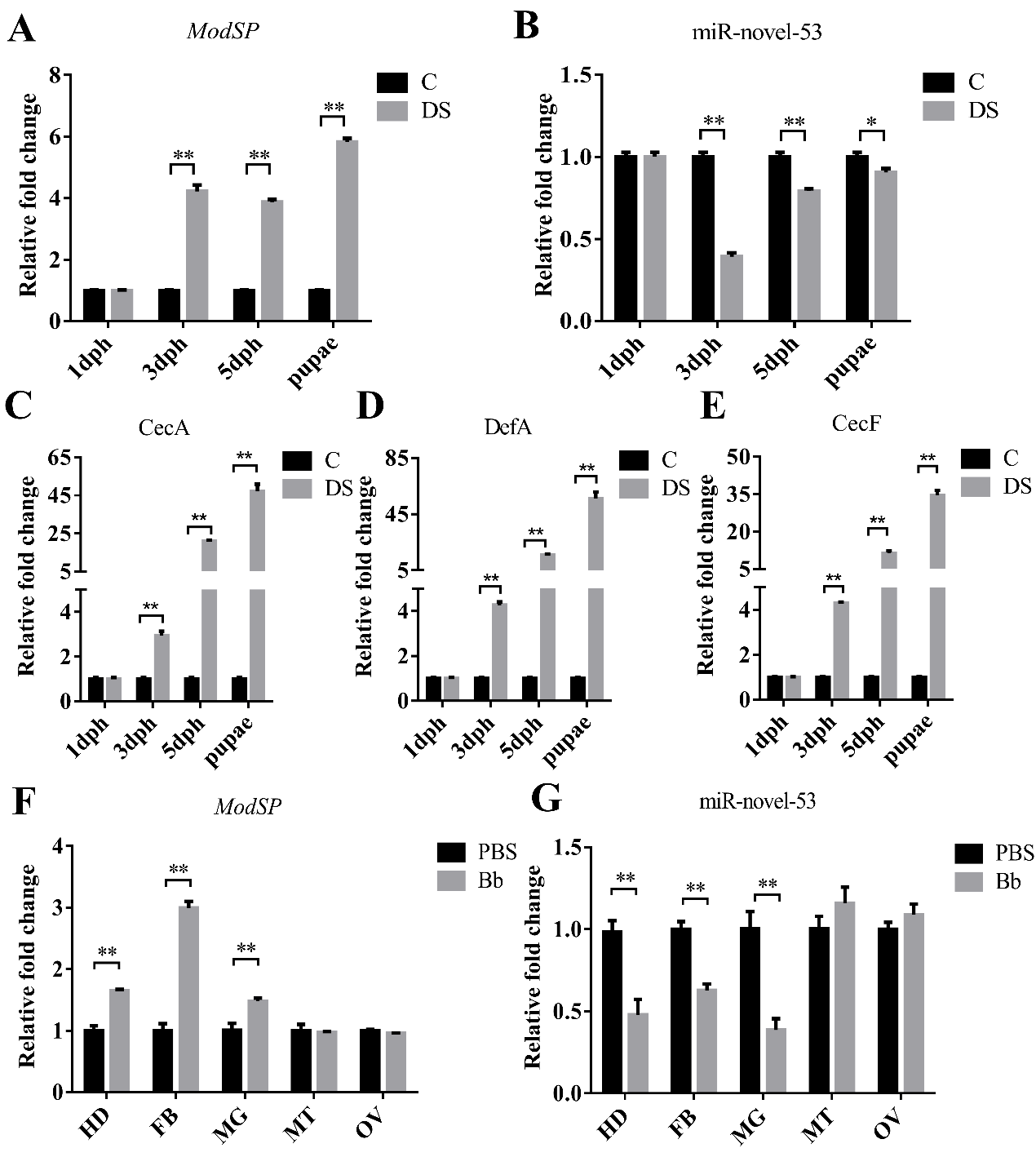


**Figure S3.** Temporal-spatial expression of *ModSP* and miRNA-novel-53 in *Ae. aegypti*. The relative mRNA abundance of *ModSP* (A) and miRNA-novel-53 abundance (B) at different stages (DS) of larvae of adult *Ae. aegypti*. The relative mRNA abundance of three *AMPs* (C, D, and E) at different stages. The relative expression of *ModSP* (F) and miRNA-novel-53 (G) at different tissues of adult mosquitoes. The results were performed three times and displayed in the form of mean ± SEM, and statistical difference analysis between samples was assessed using the Student’s *t*-test. * *P* < 0.05, ** *P* < 0.01. dph, days post-hatching. HD, head. FB, fat body. MG, midgut. MT, Malpighian tubules. OV, ovary. The control group in Figure A, C, E, F, and G is mosquito larvae of 1 days post-hatching.


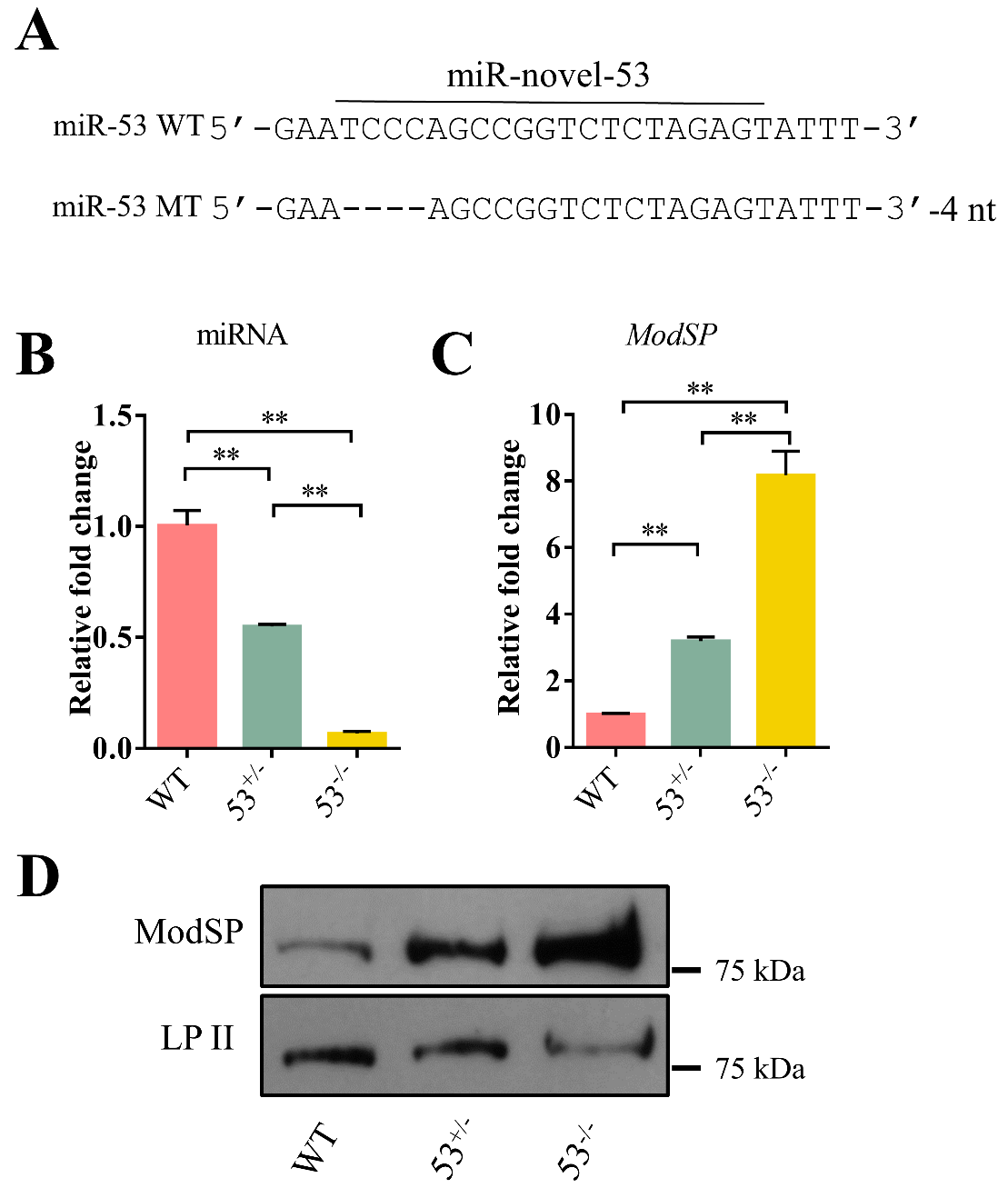


**Figure S4.** Verification of miRNA-novel-53 in *Ae. aegypti*. (A) Pairwise alignment between wild-type and the mutant-type at genome. (B) Relative quantification of miRNA in heterozygote and homozygote mosquitoes. (C) Relative quantification of *ModSP* mRNA in heterozygote and homozygote mosquitoes. (D) The abundance of ModSP protein in the knockout mosquito hemolymph. WT, wild-type mosquito; 53^+/-^, heterozygote of miRNA-novel-53 knockout; 53^-/-^, homozygote of miRNA-novel-53 knockout.


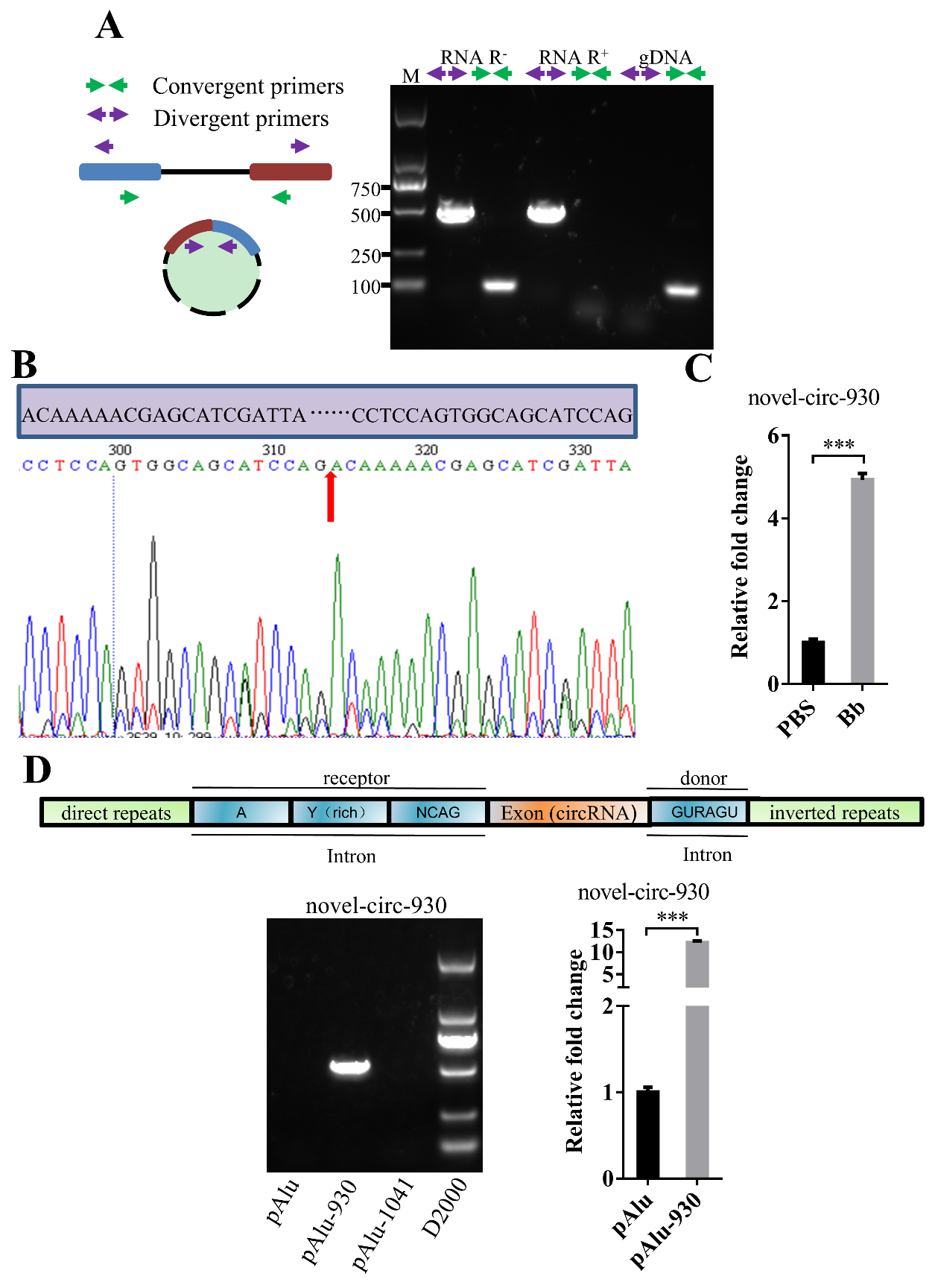


**Figure S5.** Validation and overexpression of novel-circ-930 in *Ae. aegypti*. (A) Qualitative validation of predicted circRNAs. (B) Sanger sequencing result of the validated circRNA. (C) Relative quantification of circRNAs after *B. bassiana* infection. (D) Assembly and verification of circRNA overexpression vector. (N = A, T, C, and G; R = A or C; Y = U or C). pAlu, cells transfected with pAlu5.1b empty vector; pAlu-930, transfected with pAlu5.1b-novel-circ-930 cells; pAlu-1041, transfected with pAlu5.1b-novel-circ-1041 cells. pAlu and pAlu-1041 were used as negative controls.
